# Andrastone A From the Deep-Sea-Derived Fungus *Penicillium allii-sativi* Acts as an Inducer of Caspase and RXRα-Dependent Apoptosis

**DOI:** 10.3389/fchem.2019.00692

**Published:** 2019-10-30

**Authors:** Chun-Lan Xie, Jin-Mei Xia, Ting Lin, Ying-Jie Lin, Yu-Kun Lin, Man-Li Xia, Hai-Feng Chen, Zhu-Hua Luo, Zong-Ze Shao, Xian-Wen Yang

**Affiliations:** ^1^Key Laboratory of Marine Biogenetic Resources, Third Institute of Oceanography, Ministry of Natural Resources, Xiamen, China; ^2^School of Pharmaceutical Sciences, Xiamen University, Xiamen, China

**Keywords:** deep-sea, meroterpenoids, microorganisms, anti-tumor, nuclear receptors

## Abstract

Two new (**1**, **2**) and one known (**3**) meroterpenoids were isolated from the deep-sea-derived fungus *Penicillium allii-sativi*. The relative structures of new compounds were determined on the basis of an extensive analysis of the NMR and MS data, and the absolute configurations were established by ECD calculations. Andrastone A (**1**) is a rare andrastin bearing an unusual cyclopentan-1,3-dione. It shows a selectively antiproliferative effect against HepG2 tumor cells with an IC_50_ value of 7.8 μM. Mechanism study showed that apoptosis via Caspase and RXRα pathways are responsible for the inhibitory effect.

## Introduction

**Figure 1:**
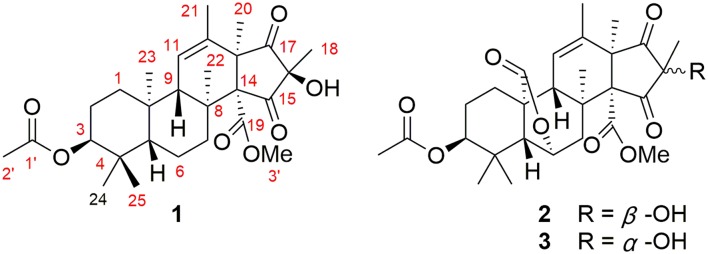
Chemical structures for compounds **1**–**3**.

The ocean yields an impressive array of novel compounds with exquisite structures and potent bioactivities (Blunt et al., 2017, 2018). Interestingly, more and more evidence shows that marine natural compounds are mainly produced by marine microorganisms. In 2017, 57% of the total new marine natural products were reported from marine microorganisms (Carroll et al., 2019). As our research investigating bioactive secondary metabolites from the deep-sea-derived microorganisms continues, a series of interesting compounds were obtained (Yang et al., 2013; Niu et al., 2017; Xie et al., 2019). The crude extract of *Penicillium allii-sativi*, a fungus isolated from the deep-sea water of the western Pacific, showed significant antiproliferative activities against several cancer cell lines. Therefore, a chemical investigation was conducted, which led to the discovery of two new and one known meroterpenoids (Figure 1), among which compound **1** showed potent effects. Herein, we report the isolation, structure, and bioactivities of these compounds.

## Materials and Methods

### General Experimental Procedures

The NMR spectra were recorded on a Bruker 400 MHz spectrometer using TMS as an internal standard. The HRESIMS spectra were measured on a Waters Xevo G2 Q-TOF (Waters) mass spectrometer. Optical rotations were measured with an Anton Paar MCP100 polarimeter. CD spectra were measured on a JASCO J-715 spectropolarimeter. The semipreparative HPLC was carried out on an Agilent technologies 1260 infinity instrument equipped with DAD detector (Agilent, USA) using a reversed-phase C18 column (5 μm, 10 × 250 mm; Cosmosil, Japan). Column chromatography (CC) was performed on silica gel, Sephadex LH-20 (Amersham Pharmacia Biotech AB), and ODS (50 μm, Daiso, Japan).

### Fermentation, Extraction, and Isolation

*Penicillium allii-sativi* was isolated from the deep-sea water of the western Pacific at a depth of −4,302 m, in 2012. The voucher strain is preserved at the Marine Culture Collection of China (Xiamen, China) with the accession number of MCCC 3A00580.

The fungus was cultured on a PDA plate at 25°C for 3 days. The fresh mycelia were then cultured in 50 × 1 L Erlenmeyer flasks under static conditions at 28°C, each containing 80 g rice and 120 mL distilled water. After 30 days, the fermented broth was extracted with EtOAc for three times. The organic solvent was evaporated under reduced pressure to afford an organic extract (120 g). The extract was partitioned by MeOH and then extracted with petroleum ether (PE). The MeOH layer was concentrated to provide a defatted extract (60.4 g).

The extract was subjected to CC on silica gel eluted with gradient CH_2_Cl_2_-MeOH to get 8 fractions (Fr.1–Fr.8). Fractions Fr.3 (5.5 g) was CC over ODS using a gradient H_2_O-MeOH, followed by CC on silica gel (PE-acetone, 5:1) to give **1** (5.0 mg). The other fraction Fr.5 (6.7 g) was subsequently subjected to CC over ODS and Sephadex LH-20 (MeOH). Final purification by preparative HPLC using H_2_O-MeOH (20 → 80%) provided **2** (3.5 mg), and by preparative TLC (CH_2_Cl_2_-MeOH, 10:1) afforded **3** (4.3 mg), respectively.

*Andrastone A (****1****)*: colorless oil; ${\left[\alpha \right]}_{\text{D}}^{20}$ 0.32 (*c* 0.50, CHCl_3_); ${\left[\alpha \right]}_{\text{D}}^{20}$ −7.2 (*c* 0.50, MeOH); UV (MeOH) λmax (logε) 206 (3.34) nm; CD (MeOH) Δε_214_ +0.48, Δε_231_ −0.08, Δε_260_ +1.36, Δε_315_ −2.03; ^1^H and ^13^C NMR data (see Table 1), HRESIMS *m/z* 511.2672 [M + Na]^+^ (calcd for C_28_H_40_O_7_Na, 511.2666).
16-*epi*-Citreohybriddione A *(****2****)*: colorless oil; ${\left[\alpha \right]}_{\text{D}}^{20}$ −166.3 (*c* 1.0, CHCl_3_); UV (MeOH) λmax (logε) 205 (4.06) nm; CD (MeOH) Δε_215_ +5.55, Δε_222_ +2.67, Δε_254_ +14.18, Δε_310_ −36.39; ^1^H and ^13^C NMR data (see Table 1); HRESIMS *m/z* 539.2257 [M + Na]^+^ (calcd for C_28_H_36_O_9_Na, 539. 2252).
*Citreohybriddione A (****3****)*: non-merohedral twin crystal; ${\left[\alpha \right]}_{\text{D}}^{20}$ −134.5 (*c* 0.3, MeOH); ^1^H and ^13^C NMR data (see Table 1); HRESIMS *m/z* 539.2260 [M + Na]^+^ (calcd for C_28_H_36_O_9_Na, 539. 2259).

**Table 1:** ^1^H (400 MHz) and ^13^C (100 MHz) NMR spectroscopic data of compounds **1**–**3** (δ in ppm, *J* in Hz within the parenthesis).

**No**	**1^a^**		**2^b^**		**2^c^**		**3^b^**	
	***δ*_C_**	***δ*_H_**	***δ*_C_**	***δ*_H_**	***δ*_C_**	***δ*_H_**	***δ*_C_**	***δ*_H_**
1	32.6, CH_2_	1.55, m 0.99, dt (13.4, 2.7)	21.8, CH_2_	2.10 (dd, 14.0, 3.2) 1.43, dd (14.0, 5.8)	20.8, CH_2_	2.13 (d, 12.4) 1.37 m	22.1, CH_2_	2.16 (dd, 13.9, 3.2) 1.36 m
2	22.2, CH_2_	1.93, m 1.47, m	23.1, CH_2_	1.75, m 1.66 dd (13.9, 3.4)	22.0, CH_2_	1.73 m	22.9, CH_2_	1.77, m 1.67 (dd, 15.0, 5.0)
3	76.8, CH	4.55, t (2.5)	77.3, CH	4.62, br s	75.9, CH	4.63 br s	77.3, CH	4.64, d (1.8)
4	36.2, C		35.5, C		34.3, C		35.4, C	
5	48.8, CH	1.24, m	56.6, CH	1.94, s	55.2, CH	1.90 s	57.0, CH	1.91, s
6	17.0, CH_2_	1.48, m	79.4, CH	4.91, d (4.0)	77.6, CH	4.77 (d, 4.0)	79.1, CH	4.94, d (4.0)
7	31.3, CH_2_	2.60, dt (12.8, 4.7) 2.14, dt (13.1, 2.3)	36.3, CH_2_	3.07, d (14.5) 2.65, dd (13.9, 3.4)	35.0, CH_2_	2.99 (d, 14.4) 2.69 (dd, 14.5, 4.3)	36.3, CH_2_	3.04, d (14.6) 2.70, dd (14.7, 4.4)
8	38.9, C		40.8, C		39.5, C		41.1, C	
9	52.4, CH	1.79, t (1.9)	53.3, CH	2.71, t (2.1)	51.8, CH	2.59 (t, 2.1)	54.5, CH	2.42, t (2.5)
10	36.6, C		45.3, C		43.8, C		45.0, C	
11	127.2, CH	5.56, br s	125.8, CH	5.78, br s	125.3, CH	5.82 br s	125.7, CH	5.86, br s
12	131.0, C		135.1, C		133.6, C		137.5, C	
13	59.6, C		61.9, C		61.0, C		60.2, C	
14	70.8, C		74.5, C		73.1, C		75.1, C	
15	211.4, C		213.7, C		212.1, C		215.0, C	
16	71.3, C		73.0, C		71.9, C		77.7, C	
17	208.3, C		209.3, C		207.4, C		211.9, C	
18	20.2, CH_3_	1.21, s	20.0, CH_3_	1.32, s	20.1, CH_3_	1.37 s	25.7, CH_3_	1.40, s
19	167.8, C		169.2, C		167.4, C		168.9, C	
20	16.9, CH_3_	1.21, s	19.4, CH_3_	1.34, s	18.5, CH_3_	1.34 s	19.6, CH_3_	1.36, s
21	18.6, CH_3_	1.60, d (1.1)	18.8, CH_3_	1.71, s	18.7, CH_3_	1.70 s	18.7, CH_3_	1.73, s
22	18.2, CH_3_	1.18, s	21.3, CH_3_	1.40, s	20.5, CH_3_	1.41 s	20.8, CH_3_	1.39, s
23	16.5, CH_3_	0.84, s	181.1, C		178.7, C		180.6, C	
24	21.2, CH_3_	0.86, s	22.9, CH_3_	0.89, s	22.5, CH_3_	0.88 s	22.5, CH_3_	0.88, s
25	27.4, CH_3_	0.81, s	26.6, CH_3_	0.99, s	26.3, CH_3_	0.96 s	26.5, CH_3_	0.99, s
1′	170.0, C		172.2, C		170.4, C		171.8, C	
2′	21.1, CH_3_	2.03, s	20.8, CH_3_	2.04, s	21.0, CH_3_	2.02 s	20.8, CH_3_	2.01, s
3′	51.8, CH_3_	3.54, s	52.3, CH_3_	3.62, s	52.0, CH_3_	3.62 s	52.6, CH_3_	3.61, s
OH		6.37 s						

a *Measure in DMSO-d_6_*.

b *Measure in CD_3_OD*.

c *Measure in CDCl_3_*.

### X-Ray Crystallographic Analysis of Compound 3

Compound **3** was obtained as a colorless non-merohedral twin crystal. The crystal data were recorded with an Xcalibur Eos Gemini single-crystal diffractometer with Cu Kα radiation (λ = 1.54184 Å). Space group C2(5), a = 16.6584(6) Å, b = 9.2116(4) Å, c = 18.8507(10) Å, α = 90°, β = 109.545°, γ = 90°, V = 2725.97 Å^3^, Z = 4, D_calcd_ = 1.259 g/cm^3^; μ = 0.830 mm^−1^, F (000) = 1,154; The final R indices [I > 2sigma(I)] *R*_1_ = 0.0691, w*R*_2_ = 0. 2044. The absolute structure parameter was 0.2 (2). Crystallographic data of **3** have been deposited in the Cambridge Crystallographic Data Center, with deposition number CCDC 1936654. Copies of the data can be obtained, free of charge, on application to CCDC, 12 Union Road, Cambridge CB21EZ, U.K. [fax +44(0)-1233-336033; email: deposit@ccdc.cam.ac.uk].

### Anti-proliferative Assay

Cytotoxic activities of all three compounds were conducted on seven human cancer cell lines of HepG2, A549, BIU-87, BEL-7402, ECA-109, Hela-S3, and PANC-1 by MTT method as reported previously (Yang et al., 2008).

### Apoptosis Determination

After treated with **1** (5 μM) for 24 h, HepG2 cells were stained with 5 μL propidium iodide (PI) by adding 5 μL Annexin V-APC, in darkness for 15 min. The apoptosis results were analyzed using a FAC scan flow cytometer (Becton Dickinson, Sparks, Maryland, USA), as described previously (Xie et al., 2019). Paclitaxel was used as the positive control.

### Caspase-3, 8, 9 Apoptosis Assays

HepG2 cells were treated with different concentrations of compound **1** (5, 10, 20 μM) for 12 h and caspase activation was investigated using a Caspase-Glo 3/8/9 assay (Promega) following the directions provided by the kit's manufacturer. The resulting luminescence was read using a Multimode plate reader (Envision, Perkin Elmer, USA).

### RXRα Transcriptional Activity

As reported previously (Duan et al., 2010), the two target plasmids (30 ng pBind RXRα LBD and 60 ng PG5 LUC) were transfected by Liposome 2000 (Invitrogen) in the cell. The cells were then exposed to **1** (10, 20, 40 μM) for 16 h. According to the introduction of the Dual-Luciferase Reporter Assay System kit (promega), the activities of Firefly luciferase (FL) and Rellina luciferase (RL) were checked. The fold activities were calculated as the relative luciferase activities ratio between sample and blank control.

## Results and Discussion

Compound **1** was obtained as colorless oil. The molecular formula of C_28_H_40_O_7_ was determined according to the sodium adduct ion peak at *m/z* 511.2672 [M + Na]^+^ in its HRESIMS spectrum, indicating nine degrees of unsaturation. The ^1^H and ^13^C NMR spectra (Supplementary Material) showed 28 carbon signals including eight methyls, one methoxyl, four methylenes, four methines (one olefinic, one oxygenated, and two aliphatic), and 11 non-protonated carbons (two ketones, two carboxyls, one olefinic, one oxygenated, and five aliphatic). Except for the typical signals of one methoxyl group [δ_H_ 3.54 (3H, s); δ_C_ 51.8 q] and one acetoxyl moiety [δ_H_ 2.03 (3H, s); δ_C_ 21.1 q, 170.0 s] group, the remaining signals indicated a 25-carbon skeleton of **1**. Since two ketones, two carboxyls, and one olefinic moiety accounted for five degrees of unsaturation, compound **1** should be a tetracyclic molecule.

**Figure 2:**
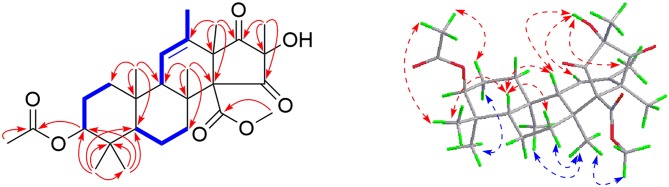
Selected COSY (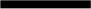), HMBC (
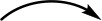
), and NOESY (
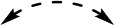
) correlations of **1**.

In the COSY spectrum, three fragments were easily deduced according to the correlations of H-3/H-2/H-1, H-5/H-6/H-7, and H-9/H-11/H-21. In the HMBC spectrum, the long-range cross peaks originated from Me-18, Me-20, and Me-22–Me-25 established a tetracyclic skeleton of **1**. Further HMBC correlations of H-3 and H-3′ to the carboxyl groups at δ_C_ 170.0 and 167.8 confirmed the attachment of the acetoxyl and methoxyl at C-3 and C-19, respectively. Accordingly, the planar structure of **1** was established as shown in Figure 2.

The relative configuration of **1** was assigned mainly by the NOESY spectrum. Correlations were found of H-5 to H-9/Me-25/H-7a (δ_H_ 2.60, td, *J* = 12.8, 4.7 Hz), H-9 to 16-OH (δ_H_ 6.37, s), Me-25 to 3-OAc/H-2a (δ_H_ 1.47 m), H-2b (δ_H_ 1.93 m) to Me-24, H-7b (δ_H_ 2.14, td, *J* = 13.1, 2.3 Hz) to Me-22/Me-23/3′-OMe. Therefore, H-5/H-9/Me-25/3-OAc/16-OH was supposed to be in the same plane (tentatively assumed as β-orientation), which is the opposite to Me-23/H-3/Me-24/Me-22/Me-18 (regarded as α-configuration).

**Figure 3:**
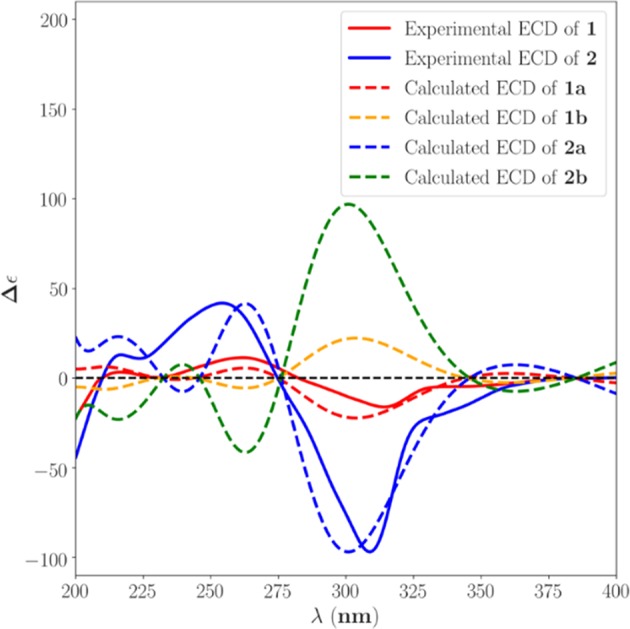
Calculated and experimental ECD spectra of **1** and **2** in MeOH.

To further assign its absolute stereochemistry, the theoretical calculation of the electronic circular dichroism (ECD) was adopted. As shown in Figure 3, the calculated ECD of (3*S*,5*S*,8*S*,9*S*,10*R*,13*R*, 14*R*,16*R*)-**1** (**a**) showed the same Cotton effects as those of the experimental ones. On the basis of the above evidence, **1** was therefore assigned and given the trivial name of Andrastone A.

**Figure 4:**
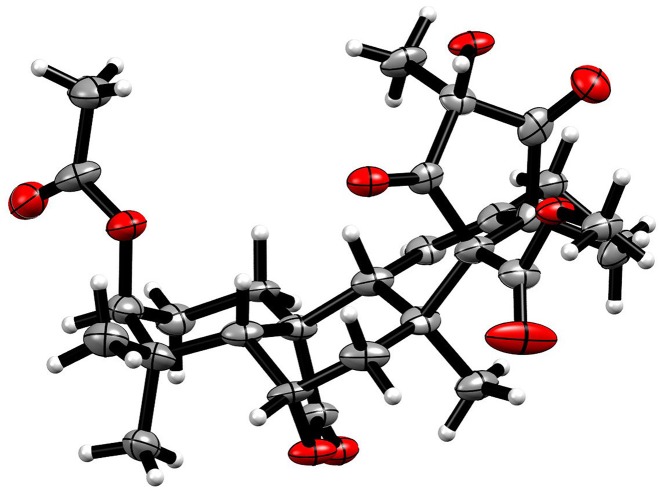
The single X-ray crystallography of **3**.

Compound **2** was also obtained as colorless oil. The molecular formula was established as C_28_H_36_O_9_ on the basis of the sodium adduct at *m/z* 539.2257 [M + Na]^+^ (calcd for C_28_H_36_O_9_Na, 539.2252) in its HRESIMS spectrum. The ^1^H and ^13^C NMR spectra showed 28 carbons consisting of seven methyls, one methoxyl, three methylenes, five methines (one olefinic, two oxygenated, and two aliphatic), and 12 non-protonated carbons (two ketones, three carboxyls, one olefinic, one oxygenated, and five aliphatic). These signals were very similar to those of compound **3**, a meroterpene that was established as citreohybriddione A by single X-ray crystallography (Figure 4) (Kosemura et al., 1991a). However, clear differences were found in **2** showing that C-16, Me-18 were upshifted up to 4.7 and 5.7 ppm, respectively, suggesting the hydroxy moiety at the C-16 position should be the β-configuration in **2**, instead of α in **3**. This was confirmed by the NOESY correlations of 3′-Me to Me-18. By comparison of the experimental and theoretical calculation of the ECD spectra (Figure 3), **2** was therefore assigned to be 16-epi-citreohybriddione A.

Andrastone A (**1**) and 16-epi-citreohybriddione A (**2**) are two meroterpenoids biosynthesized from a sesquiterpene and a tetraketide. In fact, compounds possessing the same scaffold are rarely found in nature, including citreohybridones A and B (Kosemura et al., 1991b), Andrastins A–D (Shiomi et al., 1996; Uchida et al., 1996), atlantinones A and B (Wang et al., 2010), citreohybriddiones A–C (Kosemura, 2003), citreohybridones A–G and J–L (Kosemura, 2002), citreohybridonol (Oezkaya et al., 2018), 3-deacetylcitreohybridonol (Gao et al., 2012), 15-deacetylated citreohybridone E (Cheng et al., 2019), isocitreohybridones A–C and H–J (Kosemura, 2002, 2003), and isopenicins A–C (Tang et al., 2019). Among them, 18 possess a lactone moiety at the C-23 position. For the other 13 compounds, 10 have the keto-enol tautomerism at the cyclopentane ring. Interestingly, compound **1** is a rare meroterpene without the lactone moiety but possesses the cyclopentan-1,3-dione group.

**Figure 5:**
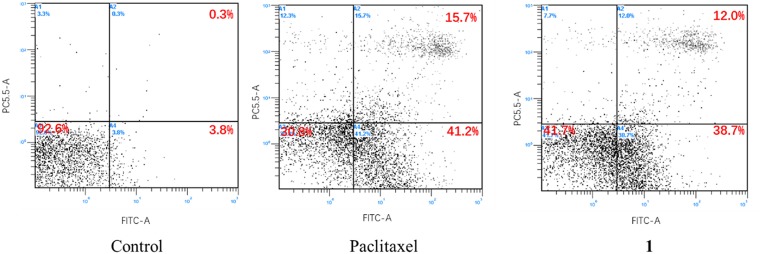
Apoptosis effect of **1** on HepG2 cells.

Compounds **1**–**3** were evaluated for their antiproliferative effects against HepG2, A549, BIU-87, BEL-7402, ECA-109, Hela-S3, and PANC-1 tumor cells. However, only compound **1** exhibited significant inhibitory activity. Interestingly, it showed a selective effect only on HepG2 cells with an IC_50_ value of 7.8 μM. To uncover its inhibition mechanism, **1** was first subjected to flow cytometry. According to a previous study, Paclitaxel can induce apoptosis via arresting cells mainly in the G2/M phase of the cell cycle (Jelinek et al., 2015; Maushagen et al., 2016). Therefore, it was used as the positive control. As shown in Figure 5, Paclitaxel and **1** could obviously induce apoptosis in HepG2 cells.

**Figure 6:**
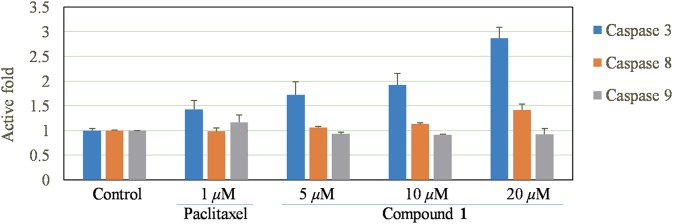
Effect of compound **1** on caspase-3, 8, 9 signaling pathways.

Since caspase is a key pathway to induce apoptosis (Porter and Janicke, 1999; Li and Yuan, 2008), we measured the activities of the caspase-3, 8, and 9. As a result, **1** could significantly increase the activities of caspase-3 and caspase-8, but had almost no effect on caspase-9 (Figure 6), suggesting that the apoptosis was caused by direct caspase-8-mediated caspase-3 activation (Kaufmann et al., 2008).

**Figure 7:**
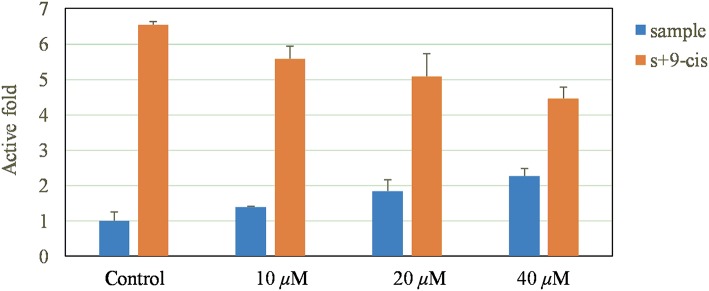
Promoting effect of **1** on reporter transcription activities of RXRα.

Moreover, a dual luciferase reporter gene assay was also carried out to investigate the RXRα transcriptional activity. Interestingly, **1** not only increased the reporter transcriptional activation of RXRα, but also reduced the transactivity of RXRα induced by 9-cis-RA (Figure 7).

## Conclusions

In summary, from the deep-sea-derived fungus *Penicillium allii-sativi*, two new and one known meroterpenes were obtained. Andrastone A (**1**) showed significant inhibitory effect selectively against HepG2 tumor cells by activating caspase-3 and regulating the transcriptional activation function of RXRα.

## Data Availability Statement

All datasets for this study are included in the manuscript/Supplementary Files.

## Author Contributions

C-LX conducted chemical and biological experiments. J-MX, Y-JL, Y-KL, and M-LX assisted C-LX's chemical experiments. TL and H-FC assisted C-LX's bioactive experiments. Z-HL isolated the fungus and Z-ZS deposited it to MCCC. X-WY initiated and oversaw all research.

### Conflict of Interest

The authors declare that the research was conducted in the absence of any commercial or financial relationships that could be construed as a potential conflict of interest.
