# Supplementary material for: Andrastone A From the Deep-Sea-Derived Fungus Penicillium allii-sativi Acts as an Inducer of Caspase and RXRα-Dependent Apoptosis
Source: Front Chem. 2019 Oct 30;7:692. doi: 10.3389/fchem.2019.00692 (PMC6833938; doi:10.3389/fchem.2019.00692)
Supplement: Supplementary file 1 [file Data_Sheet_1.PDF]

Supplementary Material

1. Computational methods

1.1. Conformational analysis

Conformational analysis was initially performed using RDKit Toolkit by Genetic Algorithm at MMFF94 force field within 10 kcal/mol with RMSD threshold of 0.25 Å for configuration **a** of compounds **1** and **2** (**Scheme S1**). The energies of all conformers were provided in **Table S1**.

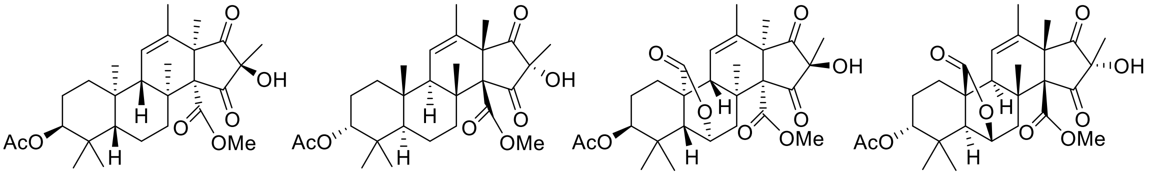

Scheme S1 Chemical structure of the enantiomers **a** and **b** of compounds **1** and **2**.

1.2. ECD calculation

The theoretical calculations were carried out using Gaussian 09 (M. J. Frisch, 2009). At first, all conformers were consecutively optimized at PM6 and HF/6-31G(d) levels. Dominative conformers with energy within 3 kcal/mol to the lowest were selected and finally optimized at B3LYP/6-311G(d,p) level. Vibrational frequency analysis confirmed the stable structures. The ECD calculations were conducted using Time-dependent Density functional theory (TD-DFT) at BP86/6-311G(d,p) level in MeOH using the IEFPCM model. Rotatory strengths for 50 excited states were calculated. The ECD spectrum was simulated in SpecDis (Bruhn et al., 2013) by overlapping Gaussian functions for each transition according to the following equation:

$$\Delta\varepsilon(E) = \frac{1}{2.297\times10^{-39}} \times \frac{1}{\sqrt{2\pi\sigma}} \sum_{i}^{A} \Delta E_i R_i e^{-\left(\frac{E-E_i}{2\sigma}\right)^2}$$

where σ represents the width of the band at 1/*e* height, and ΔE_i and R_i are the excitation energies and rotatory strengths for transition *i*, respectively.

The σ and UV-shift values were set 0.30 eV and −30 nm, respectively. The spectrum of the enantiomer **b** is derived from a by mirror inversion operation.

References

RDKit: Open-Source Cheminformatics Software [Online]. Available: http://www.rdkit.org/ [Accessed]

Bruhn, T., Schaumloffel, A., Hemberger, Y., and Bringmann, G. (2013). SpecDis: quantifying the comparison of calculated and experimental electronic circular dichroism spectra. *Chirality* 25, 243–249. doi: 10.1002/chir.22138

M. J. Frisch, G.W.T., H. B. Schlegel, G. E. Scuseria, M. A. Robb, J. R. Cheeseman, G. Scalmani, V. Barone, B. Mennucci, G. A. Petersson, H. Nakatsuji, M. Caricato, X. Li, H. P. Hratchian, A. F. Izmaylov, J. Bloino, G. Zheng, J. L. Sonnenberg, M. Hada, M. Ehara, K. Toyota, R. Fukuda, J. Hasegawa, M. Ishida, T. Nakajima, Y. Honda, O. Kitao, H. Nakai, T. Vreven, J. A. Montgomery, Jr., J. E. Peralta, F. Ogliaro, M. Bearpark, J. J. Heyd, E. Brothers, K. N. Kudin,

V. N. Staroverov, R. Kobayashi, J. Normand, K. Raghavachari, A. Rendell, J. C. Burant, S. S. Iyengar, J. Tomasi, M. Cossi, N. Rega, J. M. Millam, M. Klene, J. E. Knox, J. B. Cross, V. Bakken, C. Adamo, J. Jaramillo, R. Gomperts, R. E. Stratmann, O. Yazyev, A. J. Austin, R. Cammi, C. Pomelli, J. W. Ochterski, R. L. Martin, K. Morokuma, V. G. Zakrzewski, G. A. Voth, P. Salvador, J. J. Dannenberg, S. Dapprich, A. D. Daniels, O. Farkas, J. B. Foresman, J. V. Ortiz, J. Cioslowski, D. J. Fox. (2009). "Gaussian 09 Revision D.01". (Wallingford, CT)

2. Energies and Coordinates

2.1. Energies at MMFF94 force field

Systematic search was performed by RDKit toolkit at MMFF94 force field. Conformers were obtained with filtration by RMSD threshold of 0.25 Å and energy window of 10 kcal/mol.

Table S1 Energies at MMFF94x force field.

Configuration	Conformer	E (kcal/mol)	ΔE (kcal/mol)	Configuration	Conformer	E (kcal/mol)	ΔE (kcal/mol)
1a	1	124.78	0.00	2a	1	124.39	0.00
1a	2	125.06	0.29	2a	2	124.95	0.56
1a	3	125.50	0.73	2a	3	124.96	0.57
1a	4	126.74	1.96	2a	4	125.48	1.09
1a	5	127.47	2.70	2a	5	132.34	7.96
1a	6	129.39	4.61	2a	6	132.41	8.02
1a	7	131.58	6.81	2a	7	132.48	8.09
1a	8	132.33	7.55	2a	8	132.66	8.27
1a	9	132.80	8.03	2a	9	133.00	8.61
1a	10	133.03	8.25	2a	10	133.08	8.70
1a	11	133.54	8.76	2a	11	133.25	8.86
1a	12	134.54	9.76				

2.2. Energies at BP86 theory level

Structures for ECD calculations were optimized at B3LYP/6-311G(d,p) in gas phase and energies of excited states were calculated at BP86/6-311G(d,p) in MeOH.

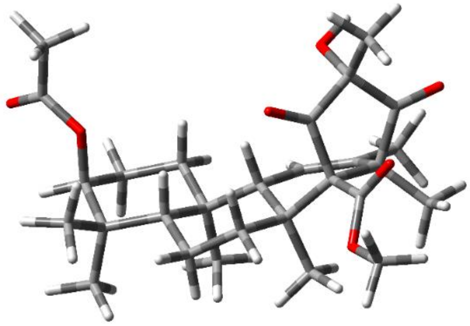

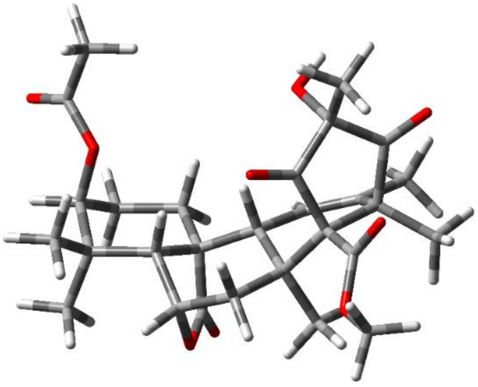

Conformer: 1a-1

SCF Energy (BP86): −1618.05042675 Hartree, −1015341.96 kcal/mol

Conformer: 2a-1

SCF Energy (BP86): −1766.1553979 Hartree, −1108279.24 kcal/mol

2.3. Coordinates at B3LYP theory level

Table S2 Standard orientations at B3LYP/6-311G(d,p) level in gas phase.

Conformer 1a-1

Center	Atomic	Atomic	Coordinates (Angstroms)		
Number	Number	Type	X	Y	Z
1	6	0	3.822730	0.490349	1.617800
2	6	0	4.370310	-0.068151	0.307660
3	6	0	3.656820	-1.357301	-0.170160
4	6	0	2.109220	-1.112180	-0.148750
5	6	0	1.502330	-0.549180	1.184500
6	6	0	2.308290	0.723479	1.565830
7	6	0	1.265660	-2.279130	-0.683420
8	6	0	-0.163270	-1.830130	-1.027260
9	6	0	-0.901840	-1.173140	0.169020
10	6	0	0.034360	-0.098820	0.827640
11	6	0	-2.182970	-0.360549	-0.280940
12	6	0	-2.975190	0.303501	0.931020
13	6	0	-2.012650	0.756841	2.043540
14	6	0	-0.690590	0.579690	1.959340
15	6	0	4.134260	-2.533461	0.709280

16	6	0	4.121430	-1.660141	-1.613730
17	8	0	4.192160	0.943549	-0.732320
18	6	0	5.183521	1.846039	-0.906730
19	8	0	6.195571	1.875029	-0.252010
20	6	0	4.847261	2.794409	-2.031780
21	6	0	1.531010	-1.523710	2.388230
22	6	0	-1.309860	-2.289240	1.164540
23	6	0	-2.625369	1.427181	3.251580
24	6	0	-4.140040	-0.520419	1.516780
25	6	0	-1.843410	0.871880	-1.215060
26	6	0	-2.664719	2.109741	-0.826630
27	6	0	-3.532569	1.619171	0.334310
28	8	0	-1.077970	0.858080	-2.139440
29	8	0	-1.715639	3.052530	-0.305980
30	6	0	-3.452299	2.696501	-1.987820
31	8	0	-4.471749	2.234771	0.775810
32	6	0	-3.231420	-1.079609	-1.146140
33	8	0	-4.115130	-0.458799	-1.698310
34	8	0	-3.118060	-2.406849	-1.243720
35	6	0	-4.104161	-3.063559	-2.072130
36	1	0	4.086380	-0.196401	2.427200
37	1	0	4.342041	1.425949	1.841320
38	1	0	5.443130	-0.249241	0.397300
39	1	0	1.972000	-0.293570	-0.867370
40	1	0	1.981130	1.108230	2.536030
41	1	0	2.100011	1.509560	0.832840
42	1	0	1.710480	-2.678070	-1.597870
43	1	0	1.254799	-3.110800	0.027830
44	1	0	-0.106690	-1.123340	-1.854830
45	1	0	-0.740800	-2.688090	-1.375790
46	1	0	0.202950	0.662540	0.056700
47	1	0	-0.092030	0.962270	2.779550
48	1	0	3.996330	-2.365381	1.775990

49	1	0	3.603329	-3.451561	0.447090
50	1	0	5.200050	-2.714521	0.539360
51	1	0	3.702880	-0.953361	-2.331470
52	1	0	5.212310	-1.597801	-1.677690
53	1	0	3.838580	-2.669771	-1.918730
54	1	0	5.624861	3.551159	-2.111540
55	1	0	4.771731	2.240889	-2.970810
56	1	0	3.878451	3.264789	-1.852280
57	1	0	1.373190	-2.564400	2.108400
58	1	0	2.481250	-1.478161	2.920620
59	1	0	0.754880	-1.259780	3.110780
60	1	0	-1.565100	-1.897810	2.148010
61	1	0	-2.158140	-2.852839	0.781700
62	1	0	-0.493621	-2.996520	1.297650
63	1	0	-3.192030	0.716221	3.861470
64	1	0	-1.842589	1.853490	3.881240
65	1	0	-3.318789	2.224641	2.973450
66	1	0	-4.722730	0.110181	2.187140
67	1	0	-4.822320	-0.855989	0.734980
68	1	0	-3.780990	-1.386059	2.072330
69	1	0	-2.221509	3.806141	0.022140
70	1	0	-4.044119	3.542091	-1.626840
71	1	0	-4.129719	1.958511	-2.417060
72	1	0	-2.749389	3.036651	-2.749770
73	1	0	-4.060840	-2.674409	-3.089170
74	1	0	-3.839991	-4.117779	-2.047650
75	1	0	-5.103890	-2.904509	-1.667620

Conformer 2a-1

Center	Atomic	Atomic	Coordinates (Angstroms)		
Number	Number	Type	X	Y	Z
1	6	0	3.856285	0.068555	1.473252
2	6	0	4.158025	0.145660	-0.022858
3	6	0	3.495695	-0.993641	-0.831068
4	6	0	1.957612	-0.932643	-0.576534
5	6	0	1.498728	-0.783508	0.899445
6	6	0	2.352763	0.148631	1.768414
7	6	0	1.163951	-2.199571	-0.918543
8	6	0	-0.316551	-1.888486	-1.164832
9	6	0	-1.006790	-1.227784	0.072072
10	6	0	0.009451	-0.277600	0.816805
11	6	0	-2.192445	-0.256969	-0.296871
12	6	0	-2.885341	0.398465	0.986370
13	6	0	-1.912553	0.538683	2.181635
14	6	0	-0.619066	0.216435	2.089550
15	6	0	4.166941	-2.326235	-0.427559
16	6	0	3.751136	-0.781112	-2.336560
17	8	0	3.631925	1.402904	-0.546560
18	6	0	4.417936	2.501648	-0.461237
19	8	0	5.517942	2.505698	0.030628
20	6	0	3.733954	3.694245	-1.083520
21	6	0	1.527241	-2.240016	1.358206
22	6	0	-1.537741	-2.370094	0.983065
23	6	0	-2.489248	1.053389	3.479448
24	6	0	-4.197483	-0.272540	1.448936
25	6	0	-1.710305	0.981590	-1.149534
26	6	0	-2.285547	2.294700	-0.606908
27	6	0	-3.186744	1.851532	0.551106
28	8	0	-1.014826	0.918080	-2.127312
29	8	0	-1.160987	2.998599	-0.054578
30	6	0	-3.003188	3.115152	-1.666511
31	8	0	-3.971725	2.587122	1.097575
32	6	0	-3.304416	-0.785916	-1.216500
33	8	0	-4.144498	-0.039806	-1.672420
34	8	0	-3.264256	-2.090048	-1.495375
35	6	0	-4.283491	-2.574016	-2.400478

36	1	0	4.275445	-0.859162	1.870327
37	1	0	4.377984	0.883057	1.980838
38	1	0	5.237057	0.146164	-0.189201
39	1	0	1.582631	-0.091098	-1.160164
40	1	0	2.182527	-0.088516	2.821107
41	1	0	2.014720	1.175920	1.606875
42	1	0	1.572480	-2.776242	-1.748338
43	8	0	1.329155	-3.038498	0.270969
44	1	0	-0.362803	-1.236953	-2.037341
45	1	0	-0.844473	-2.809123	-1.415512
46	1	0	0.133797	0.610520	0.188266
47	1	0	0.003203	0.330400	2.969718
48	1	0	4.032506	-2.592234	0.620170
49	1	0	3.776052	-3.153847	-1.022117
50	1	0	5.242530	-2.264202	-0.618797
51	1	0	3.334906	0.165557	-2.682710
52	1	0	4.824728	-0.781052	-2.547852
53	1	0	3.302660	-1.586949	-2.924590
54	1	0	4.251225	4.603847	-0.784627
55	1	0	3.774159	3.601695	-2.172425
56	1	0	2.682385	3.733521	-0.795913
57	1	0	-1.861647	-2.011460	1.956740
58	1	0	-2.376190	-2.865240	0.495749
59	1	0	-0.776255	-3.127709	1.146909
60	1	0	-3.153397	0.319762	3.946560
61	1	0	-1.685506	1.269319	4.185196
62	1	0	-3.074795	1.964607	3.331926
63	1	0	-4.677501	0.354540	2.198258
64	1	0	-4.902442	-0.371497	0.624622
65	1	0	-4.011292	-1.255334	1.882009
66	1	0	-1.514009	3.744301	0.446864
67	1	0	-3.426504	4.010168	-1.203636
68	1	0	-3.813706	2.542752	-2.119704
69	1	0	-2.284708	3.400670	-2.436135
70	1	0	-4.212257	-2.056277	-3.356825
71	1	0	-4.080614	-3.635549	-2.516432
72	1	0	-5.273544	-2.412161	-1.974397
73	8	0	1.674494	-2.678240	2.464802

3. The 1D and 2D NMR spectra of 1 and 2

Supplementary Figure 1. ^{1}H NMR spectrum of **1** in DMSO-d_6.

Supplementary Figure 2. ^{13}C NMR spectrum of **1** in DMSO-d_6.

Supplementary Figure 3. HSQC NMR spectrum of **1** in DMSO-d_6.

Supplementary Figure 4. COSY NMR spectrum of **1** in DMSO-d_6.

Supplementary Figure 5. HMBC NMR spectrum of **1** in DMSO-d_6.

Supplementary Figure 6. NOESY NMR spectrum of **1** in DMSO-d_6.

Supplementary Figure 7. ^{1}H NMR spectrum of **2** in $CDCl_3$.

Supplementary Figure 8. ^{13}C NMR spectrum of **2** in $CDCl_3$.

Supplementary Figure 9. HSQC NMR spectrum of **2** in $CDCl_3$.

Supplementary Figure 10. COSY NMR spectrum of **2** in $CDCl_3$.

Supplementary Figure 11. HMBC NMR spectrum of **2** in $CDCl_3$.

Supplementary Figure 12. NOESY NMR spectrum of **1** in $CDCl_3$.

Supplementary Figure 13. ^{1}H NMR spectrum of **2** in CD_3OD.

Supplementary Figure 14. ^{13}C NMR spectrum of **2** in CD_3OD.

Supplementary Figure 15. HSQC NMR spectrum of **2** in CD_3OD.

Supplementary Figure 16. COSY NMR spectrum of **2** in CD_3OD.

Supplementary Figure 17. HMBC NMR spectrum of **2** in CD_3OD.

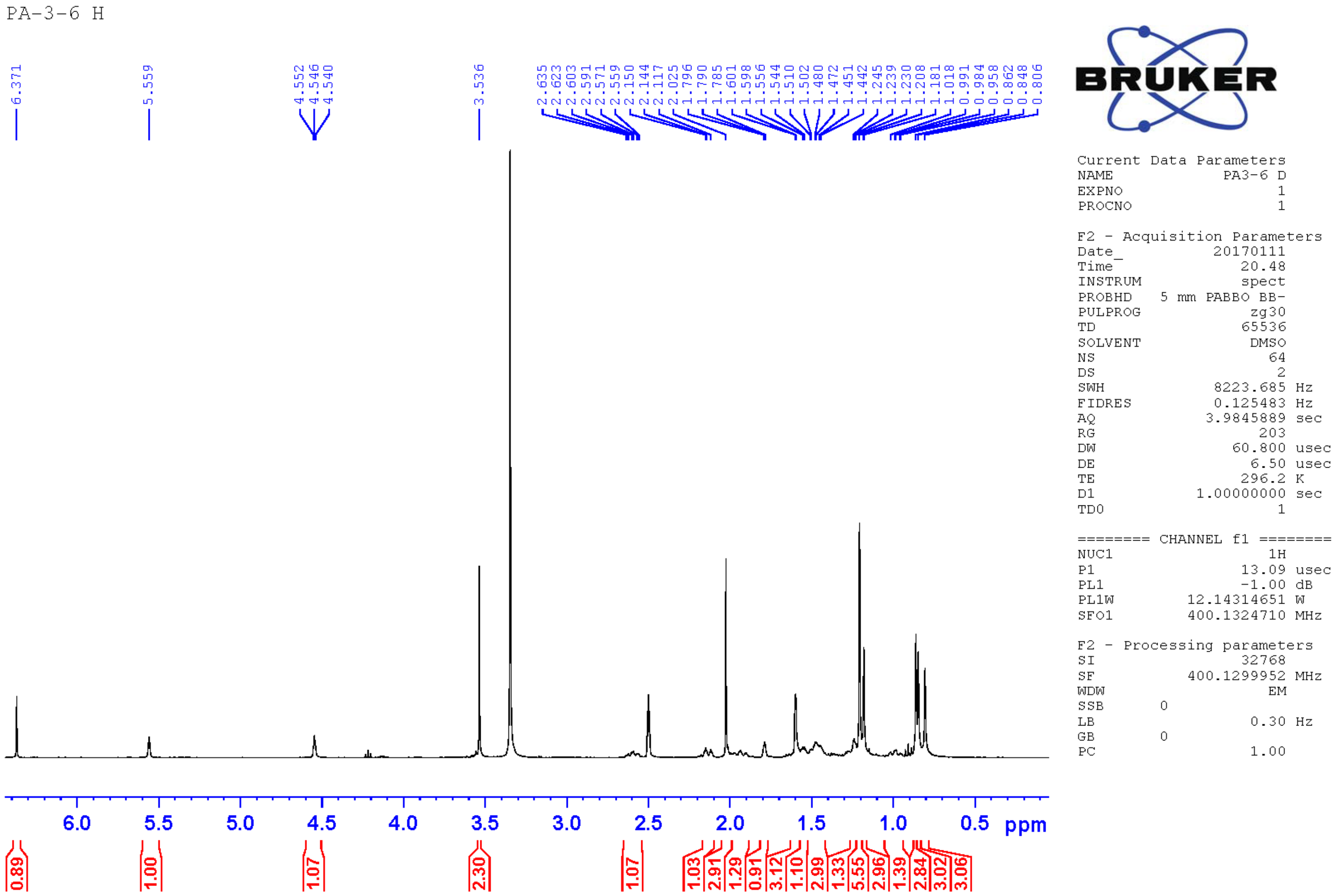

Supplementary Figure 1. ^{1}H NMR spectrum of **1** in DMSO-d_6.

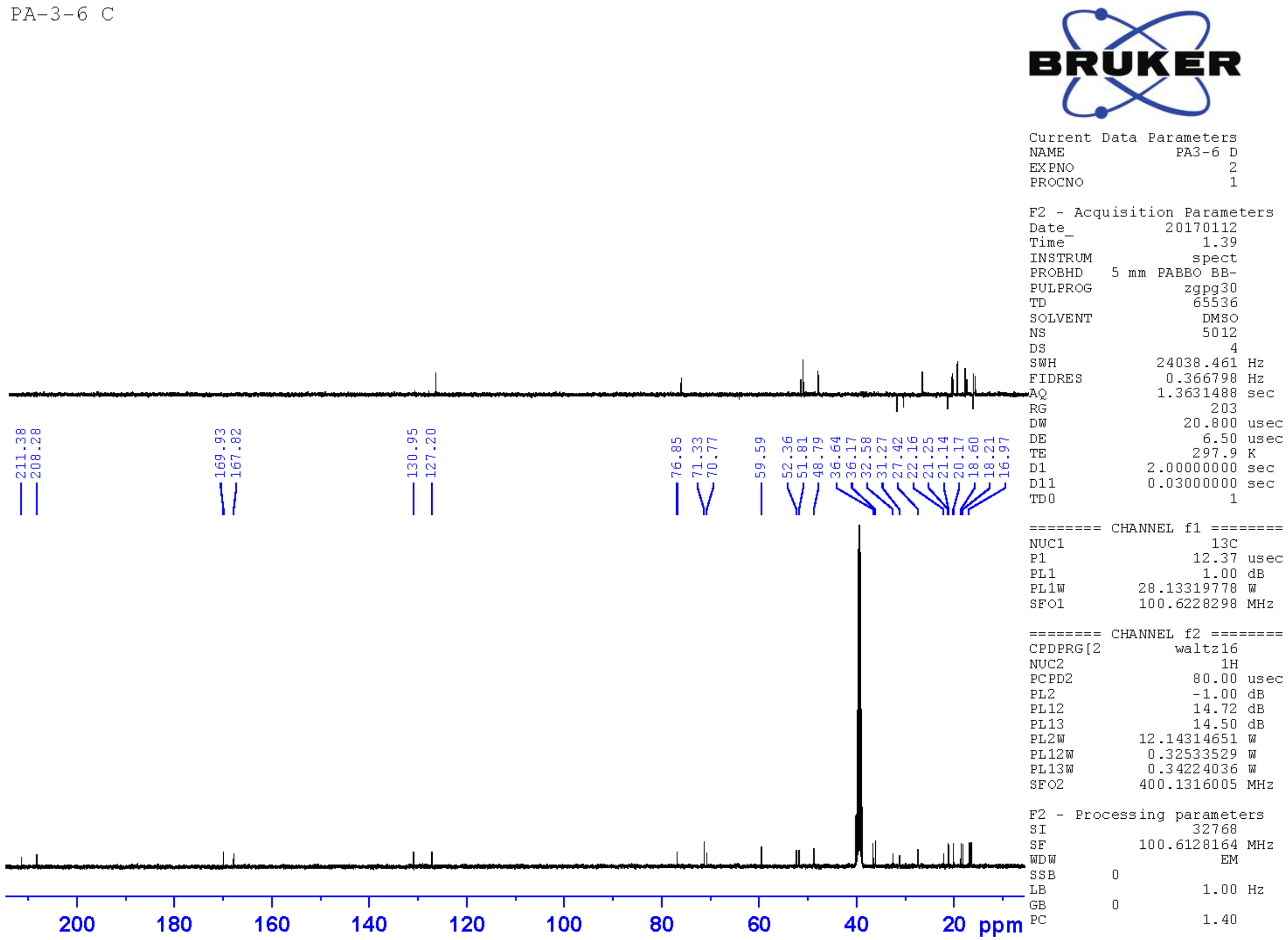

Supplementary Figure 2. ^{13}C NMR spectrum of **1** in DMSO-d_6.

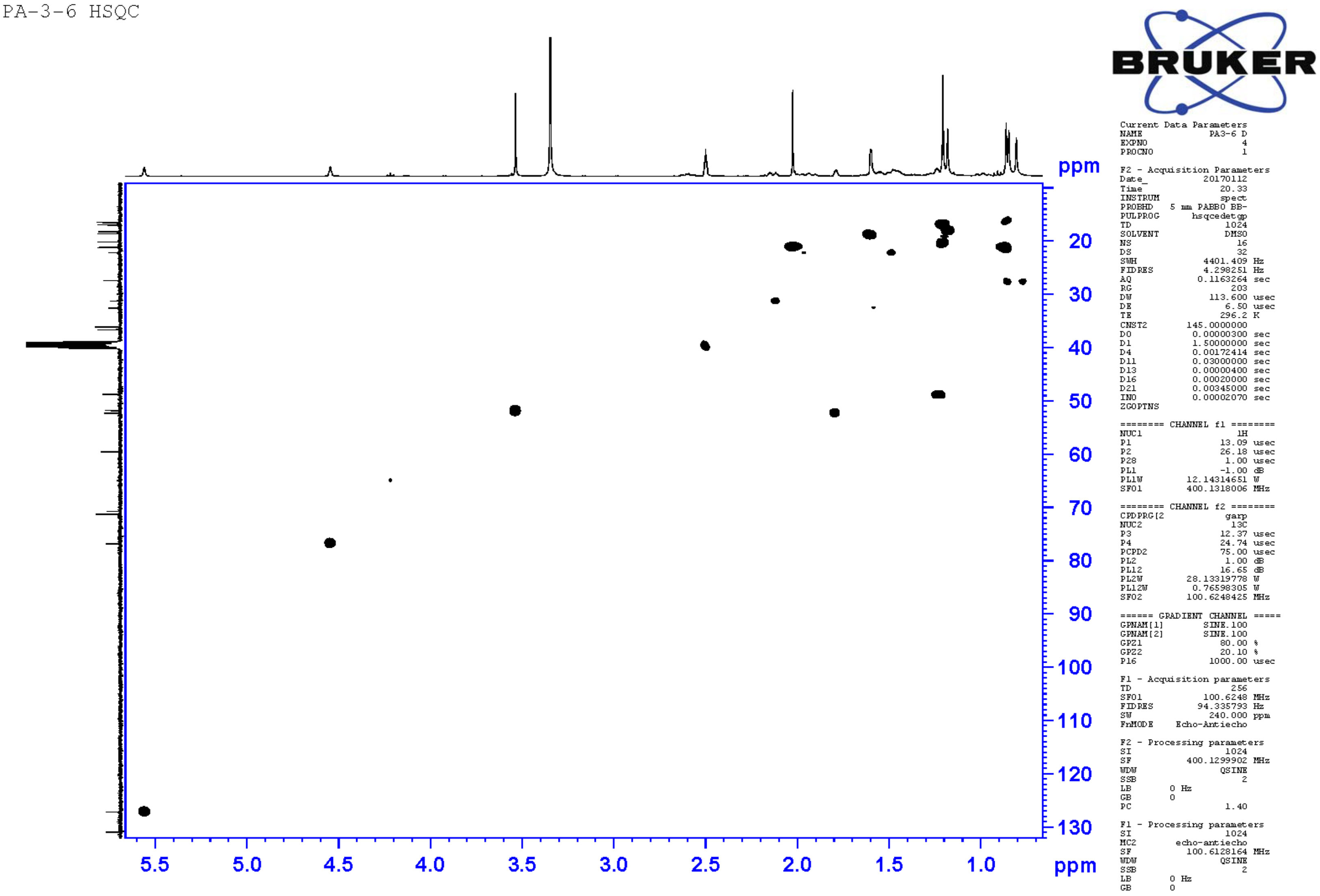

Supplementary Figure 3. HSQC NMR spectrum of **1** in DMSO-d_6.

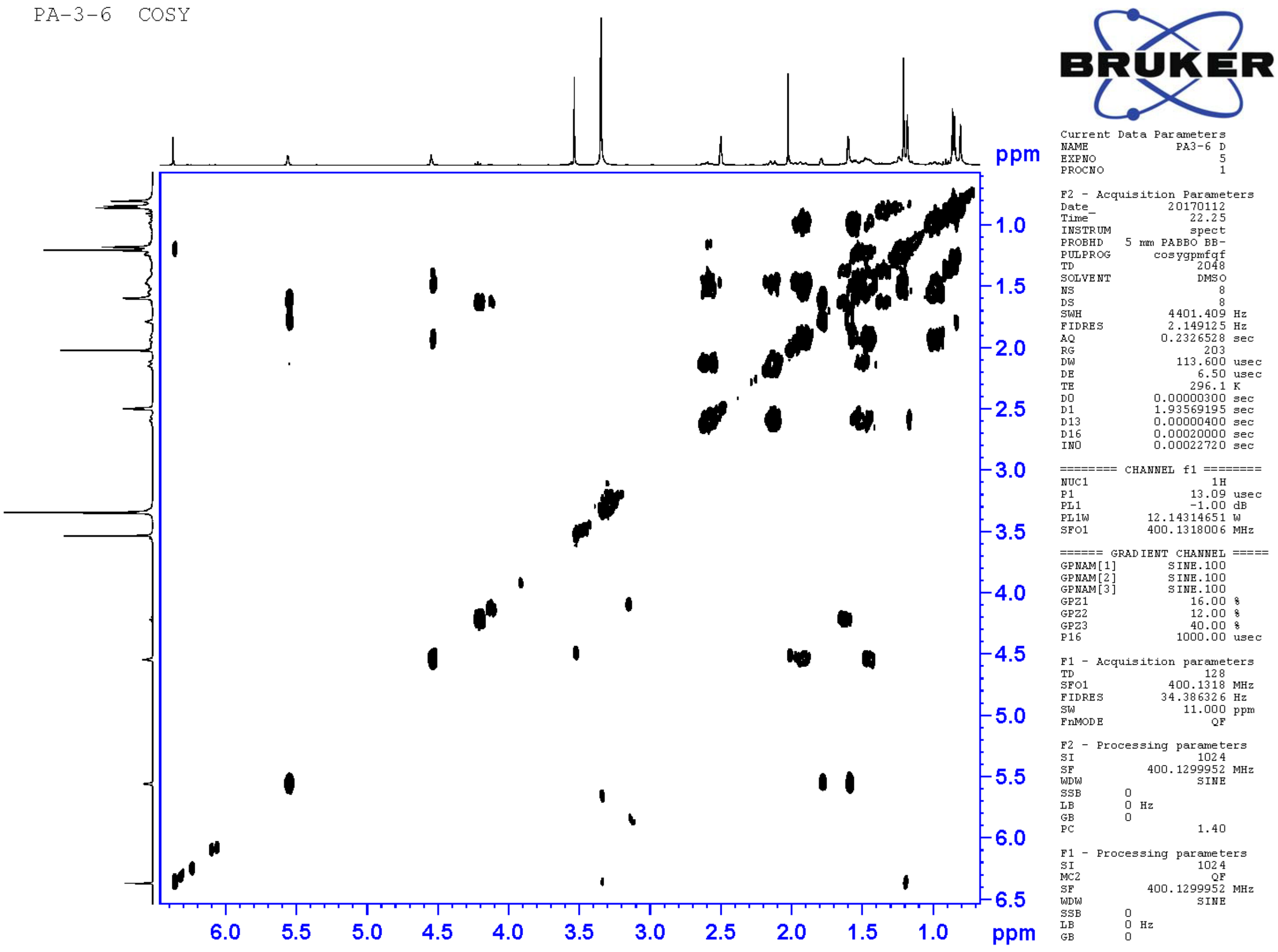

Supplementary Figure 4. COSY NMR spectrum of **1** in DMSO-d_6.

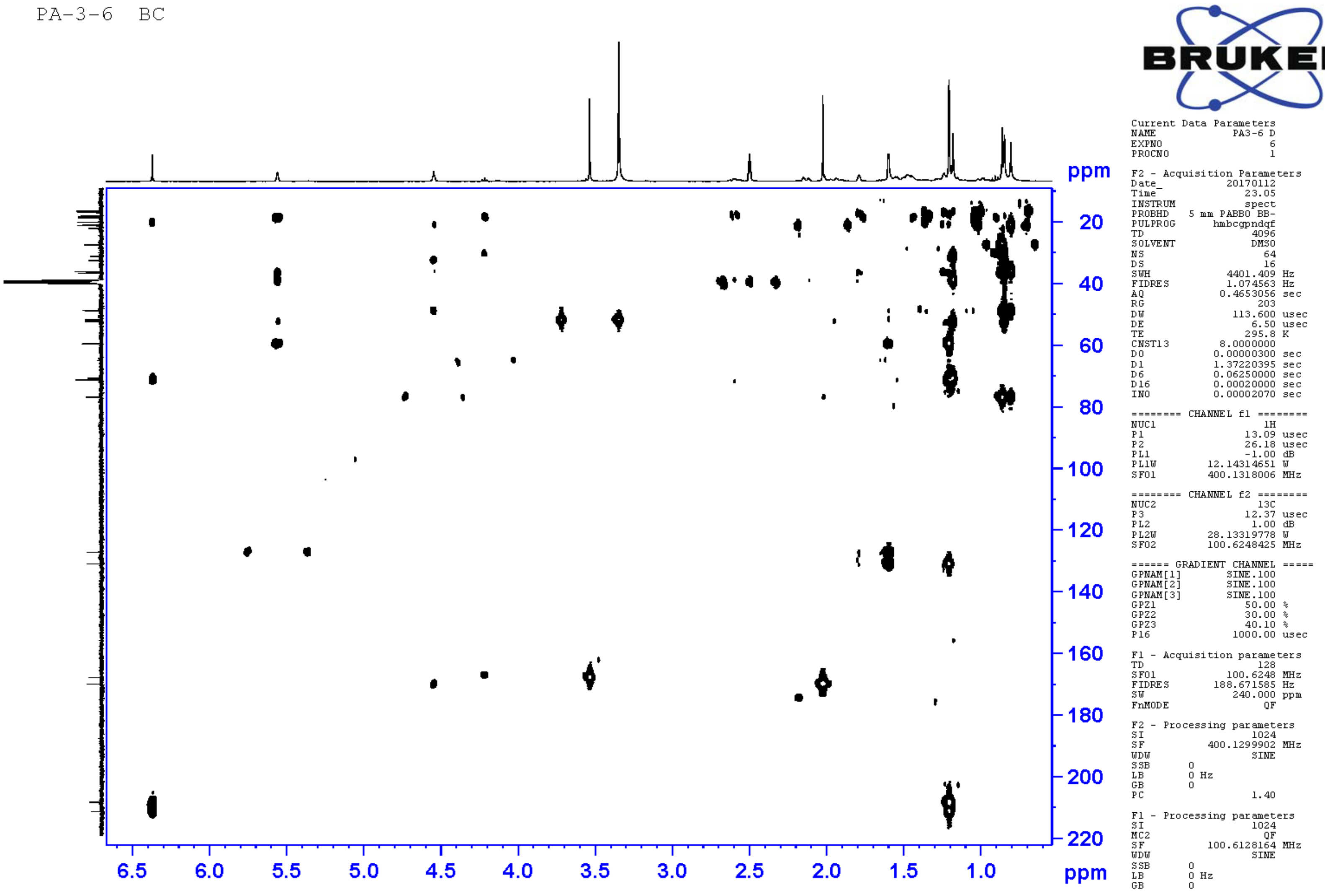

Supplementary Figure 5. HMBC NMR spectrum of **1** in DMSO-d_6.

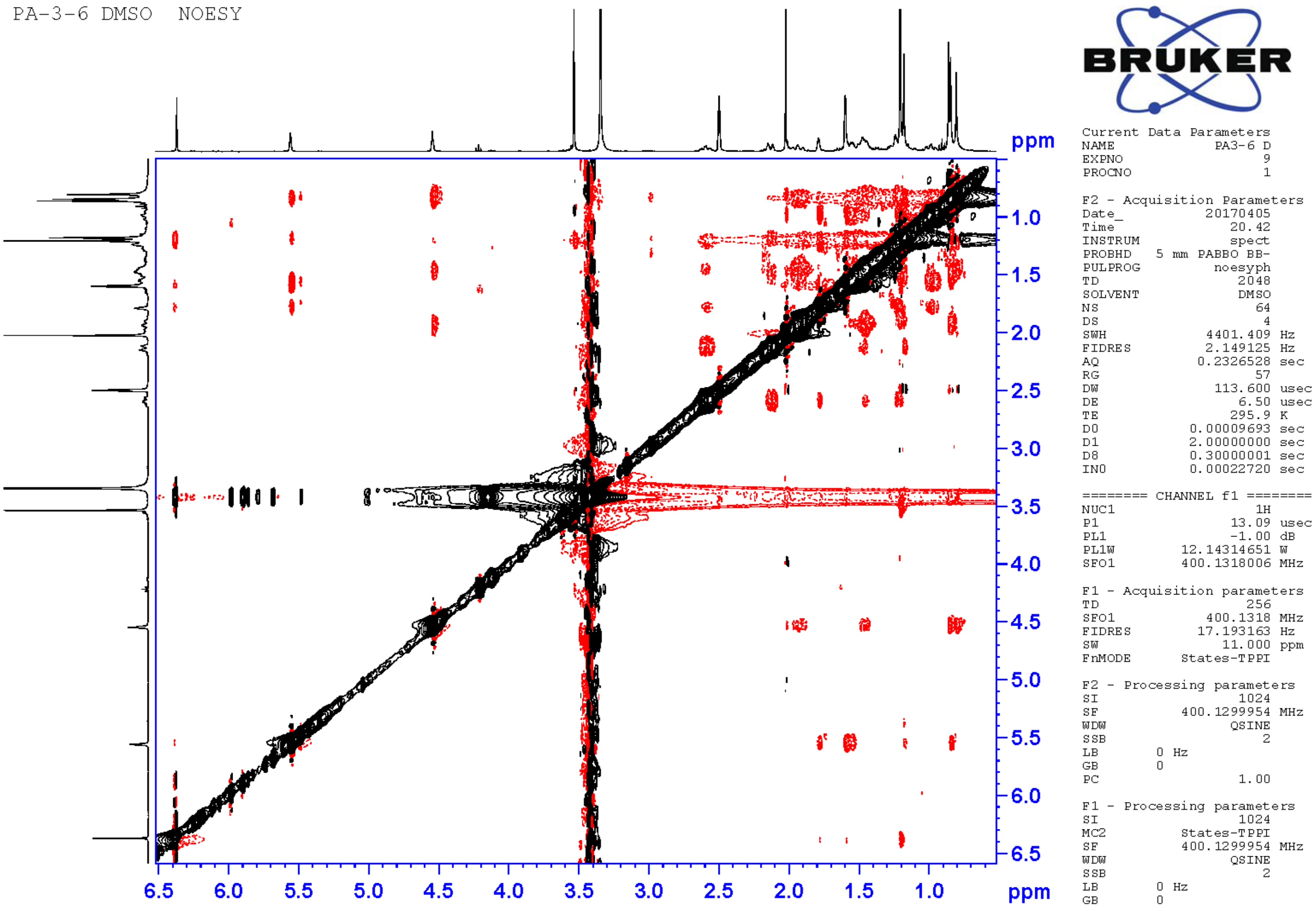

Supplementary Figure 6. NOESY NMR spectrum of **1** in DMSO-d_6.

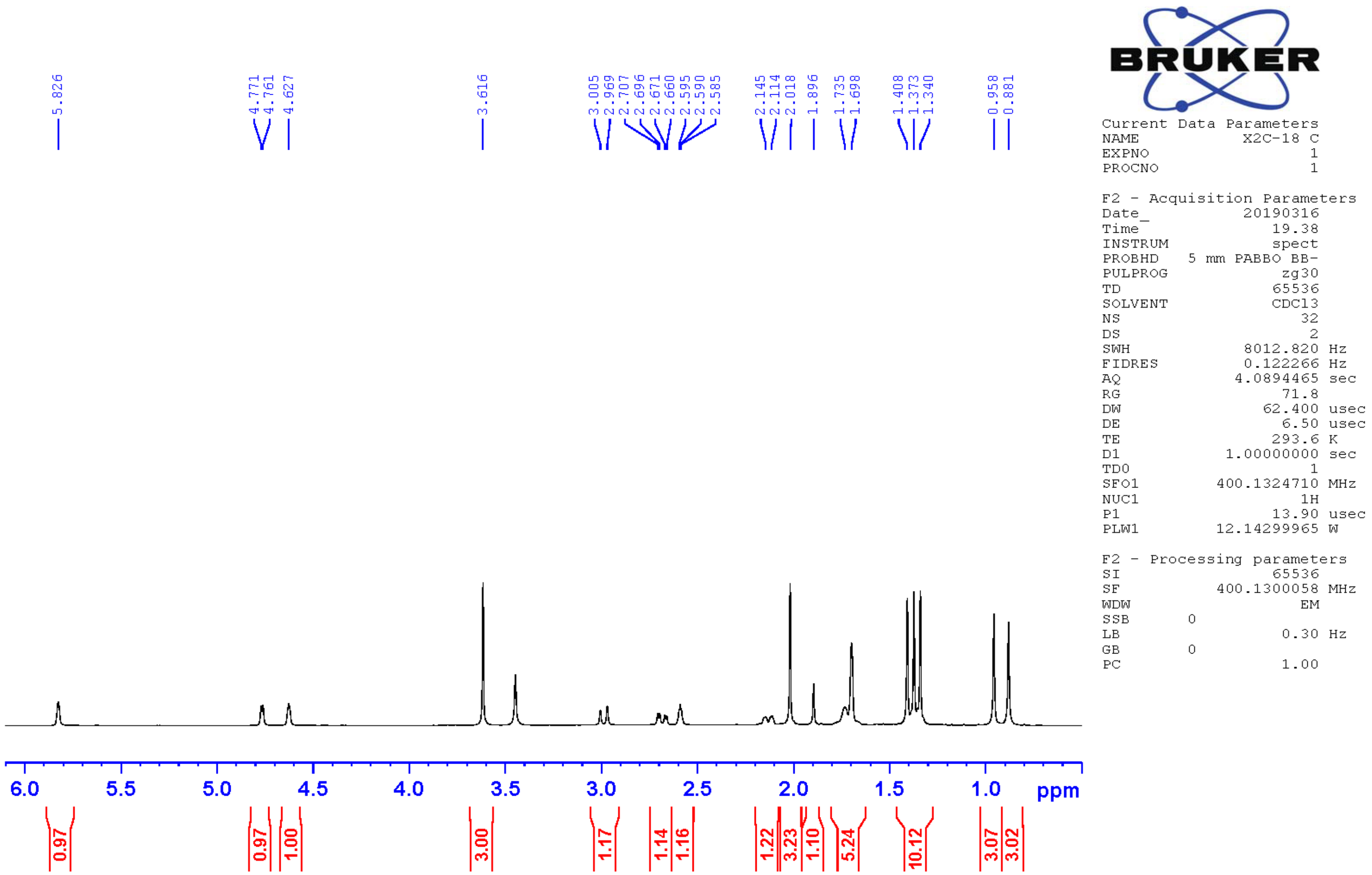

Supplementary Figure 7. ^{1}H NMR spectrum of **2** in $CDCl_3$.

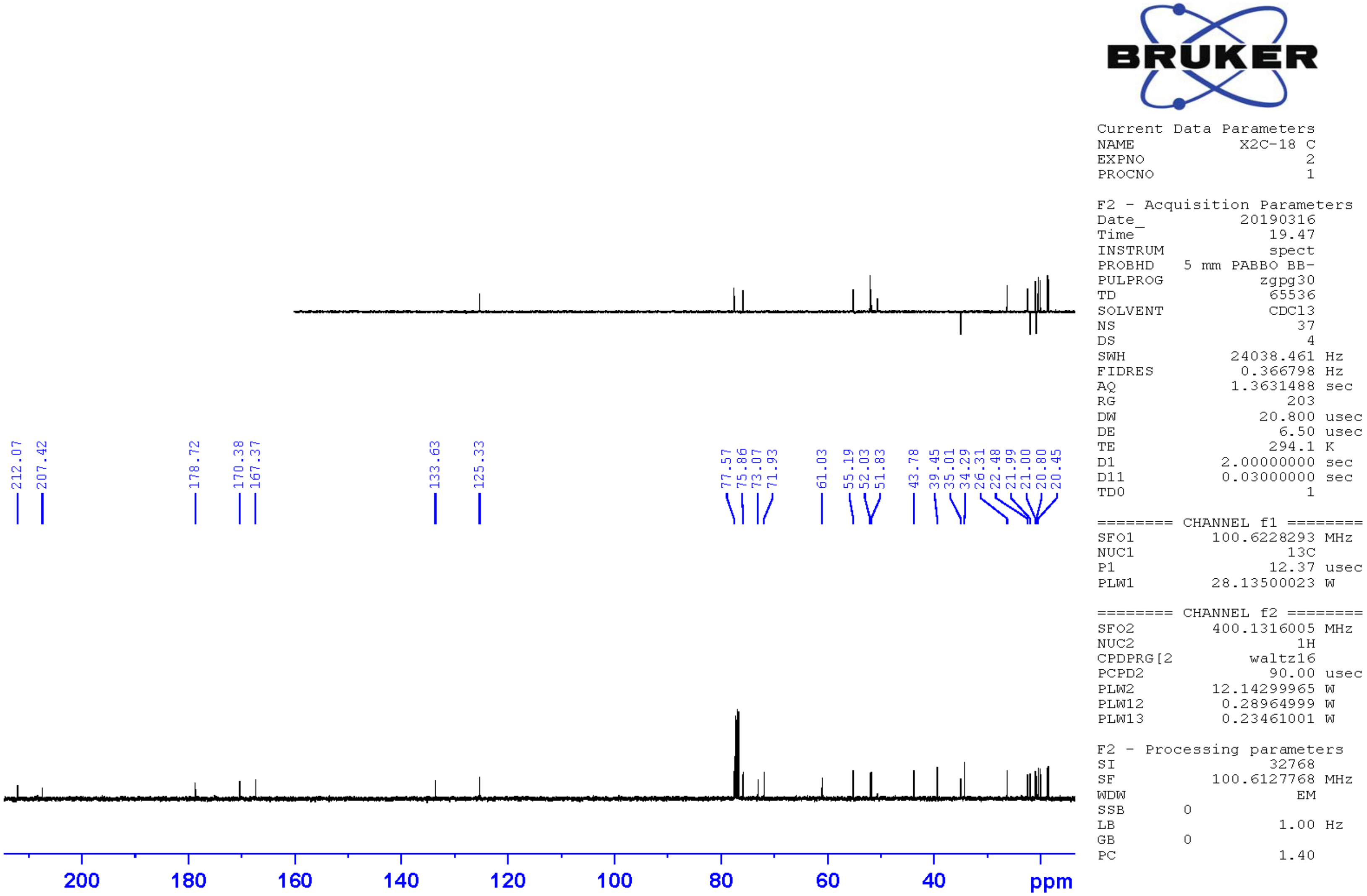

Supplementary Figure 8. ^{13}C NMR spectrum of **2** in $CDCl_3$.

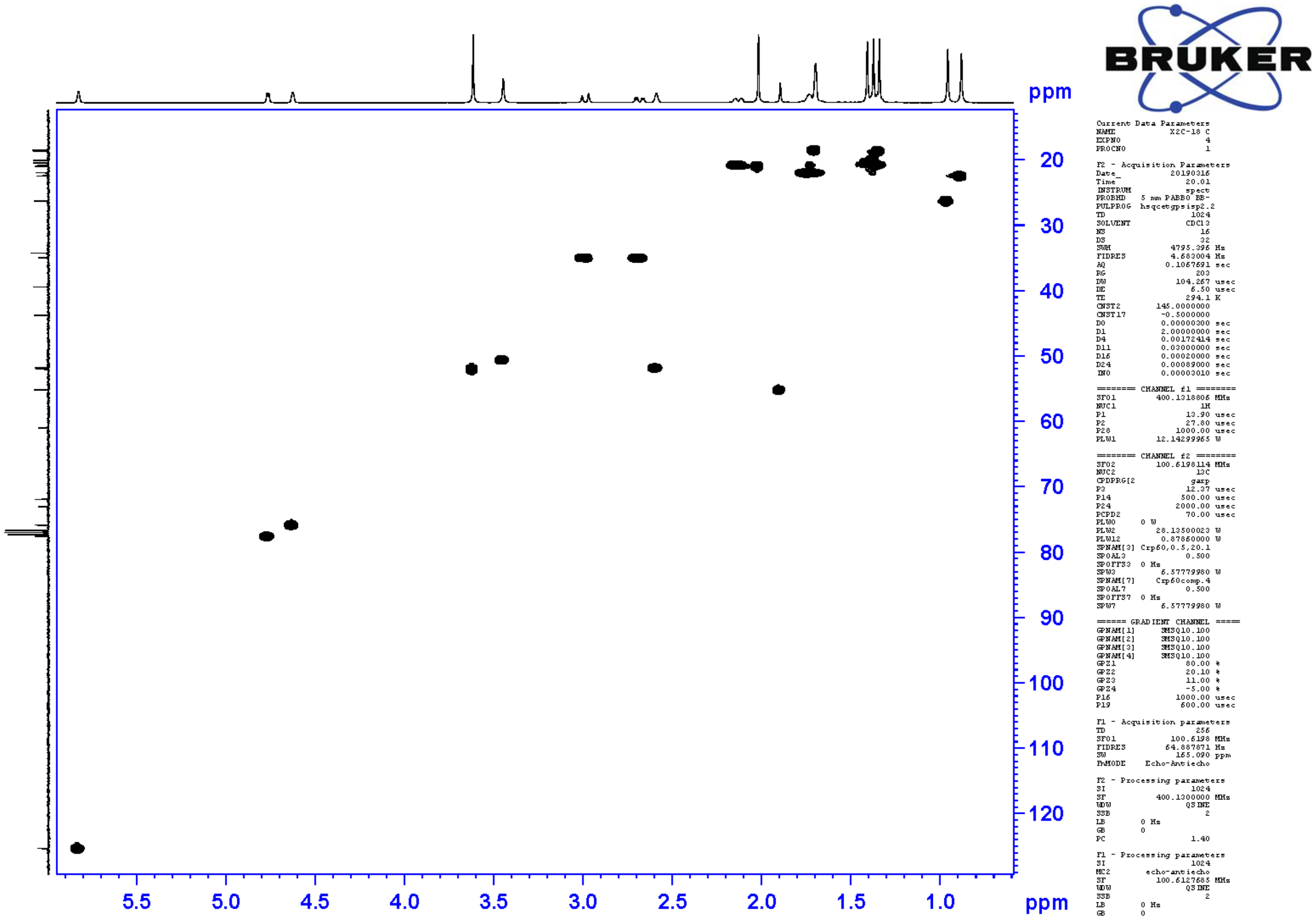

Supplementary Figure 9. HSQC NMR spectrum of **2** in $CDCl_3$.

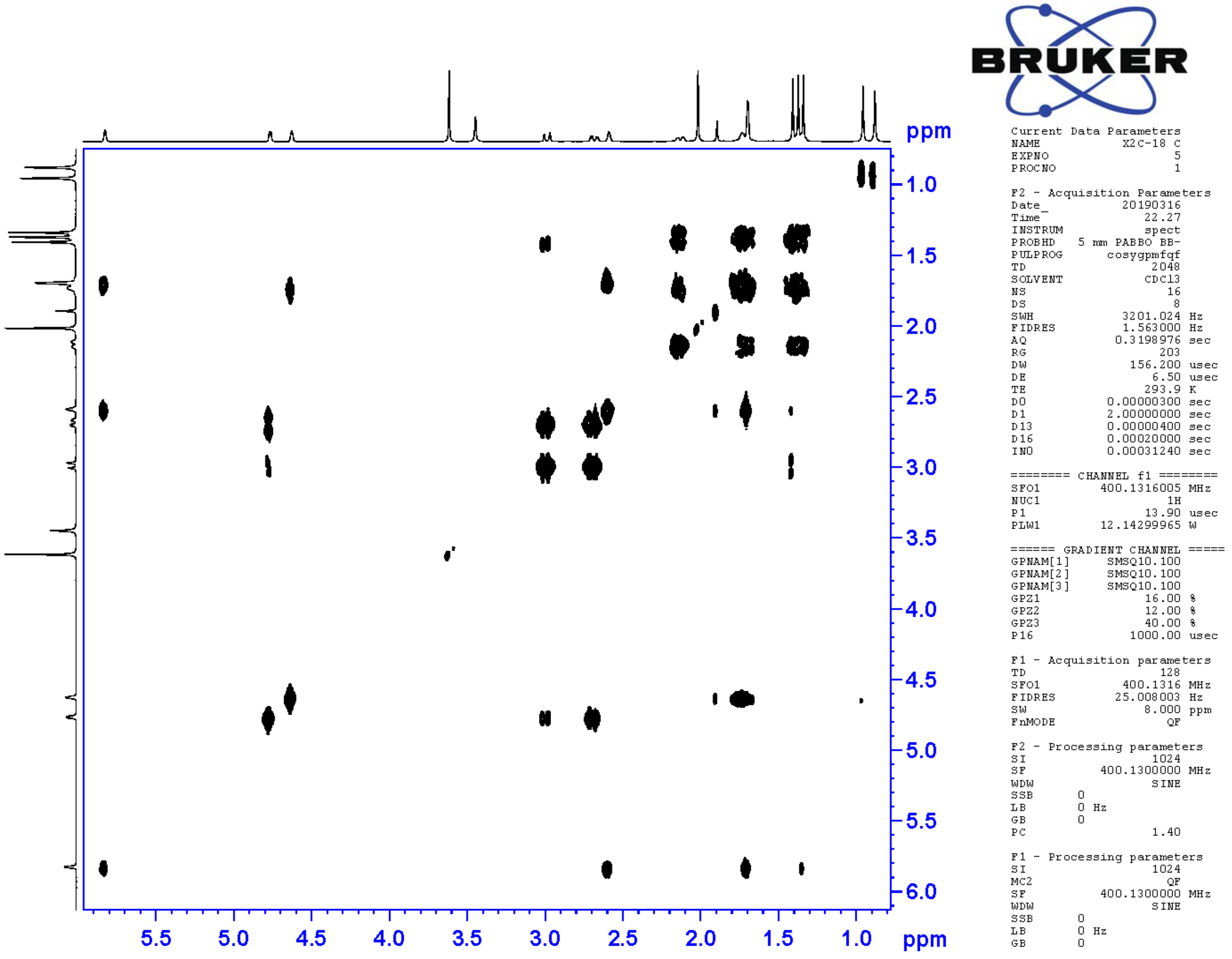

Supplementary Figure 10. COSY NMR spectrum of **2** in $CDCl_3$.

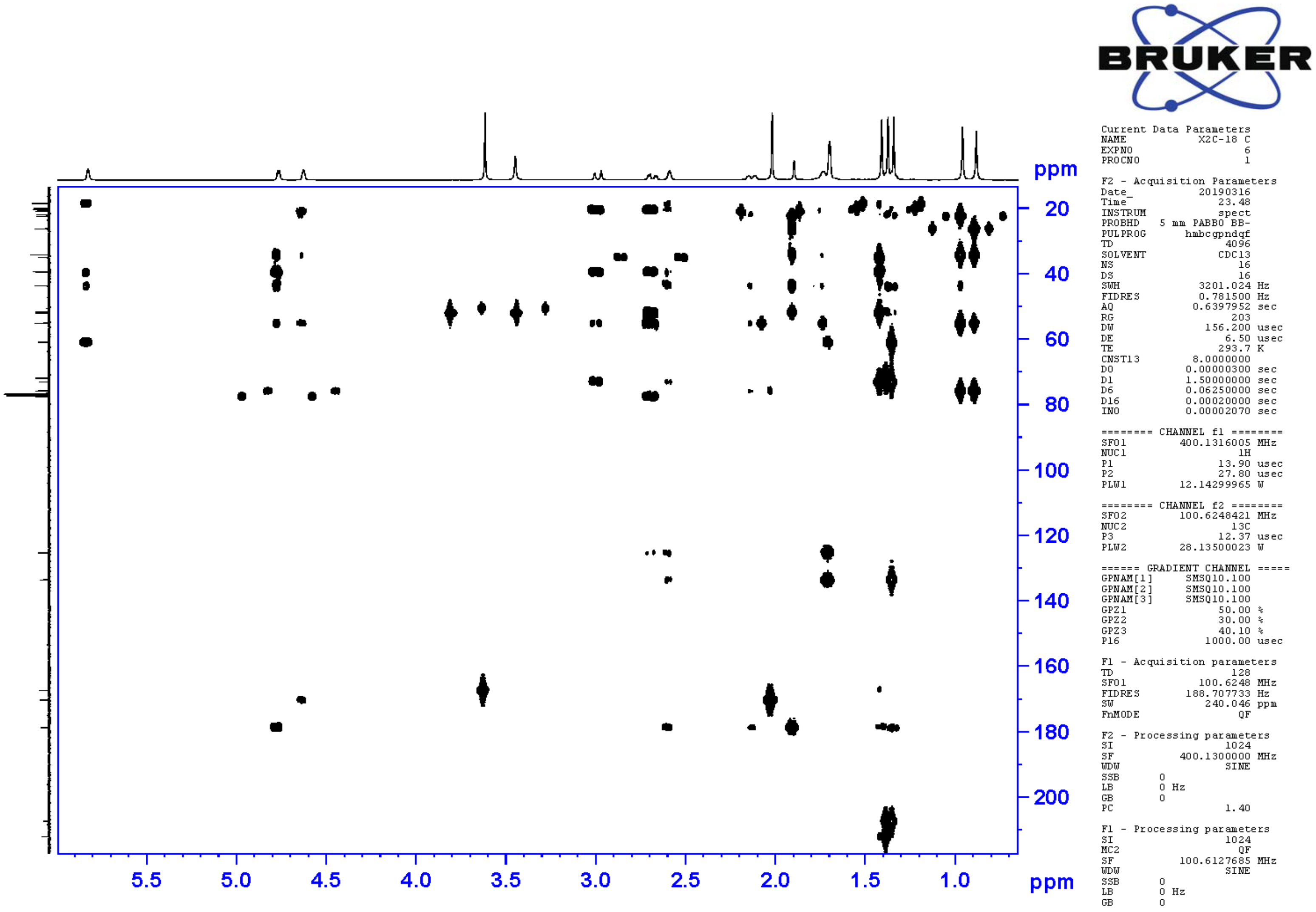

Supplementary Figure 11. HMBC NMR spectrum of **2** in $CDCl_3$.

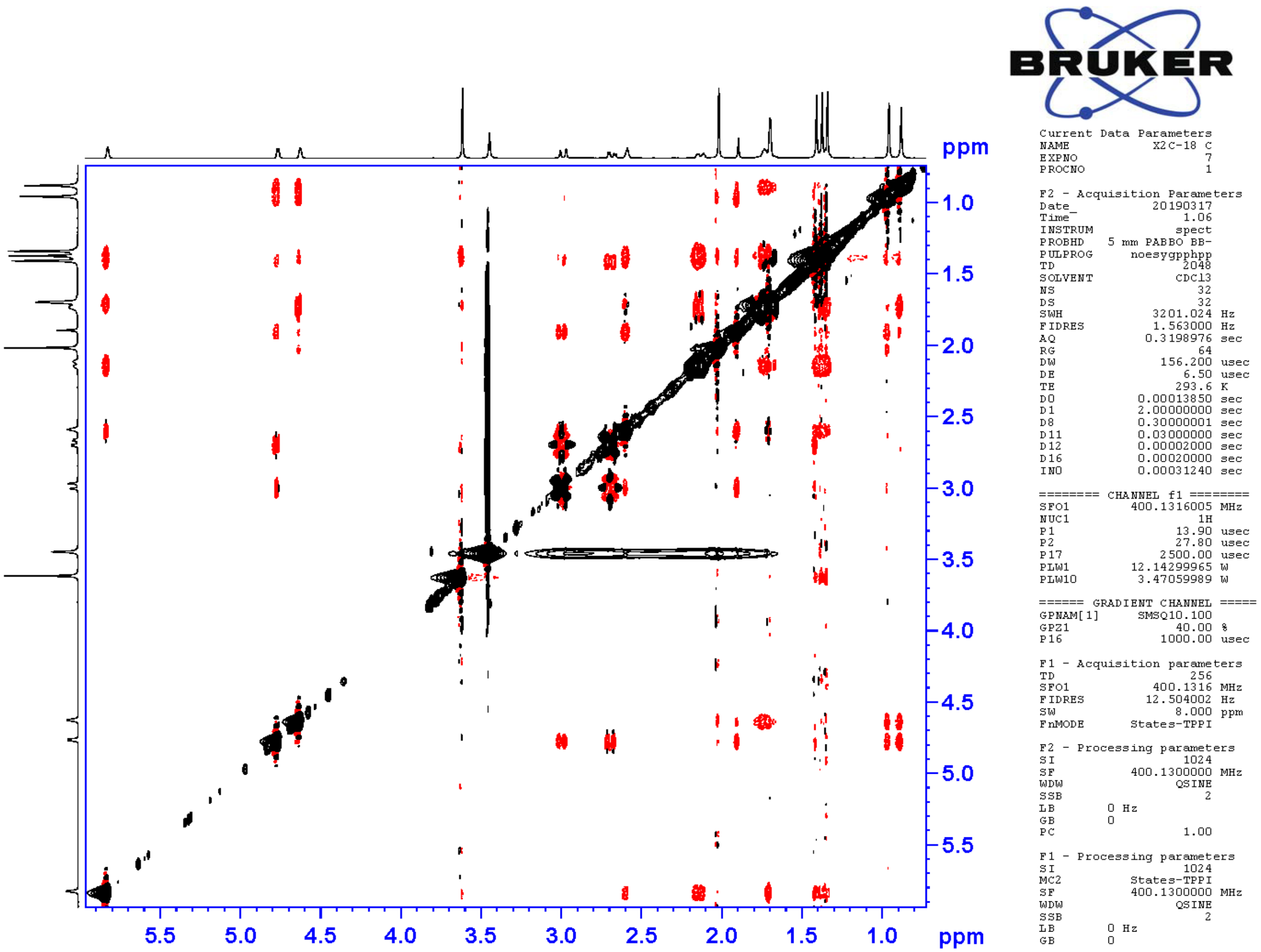

Supplementary Figure 12. NOESY NMR spectrum of **1** in $CDCl_3$.

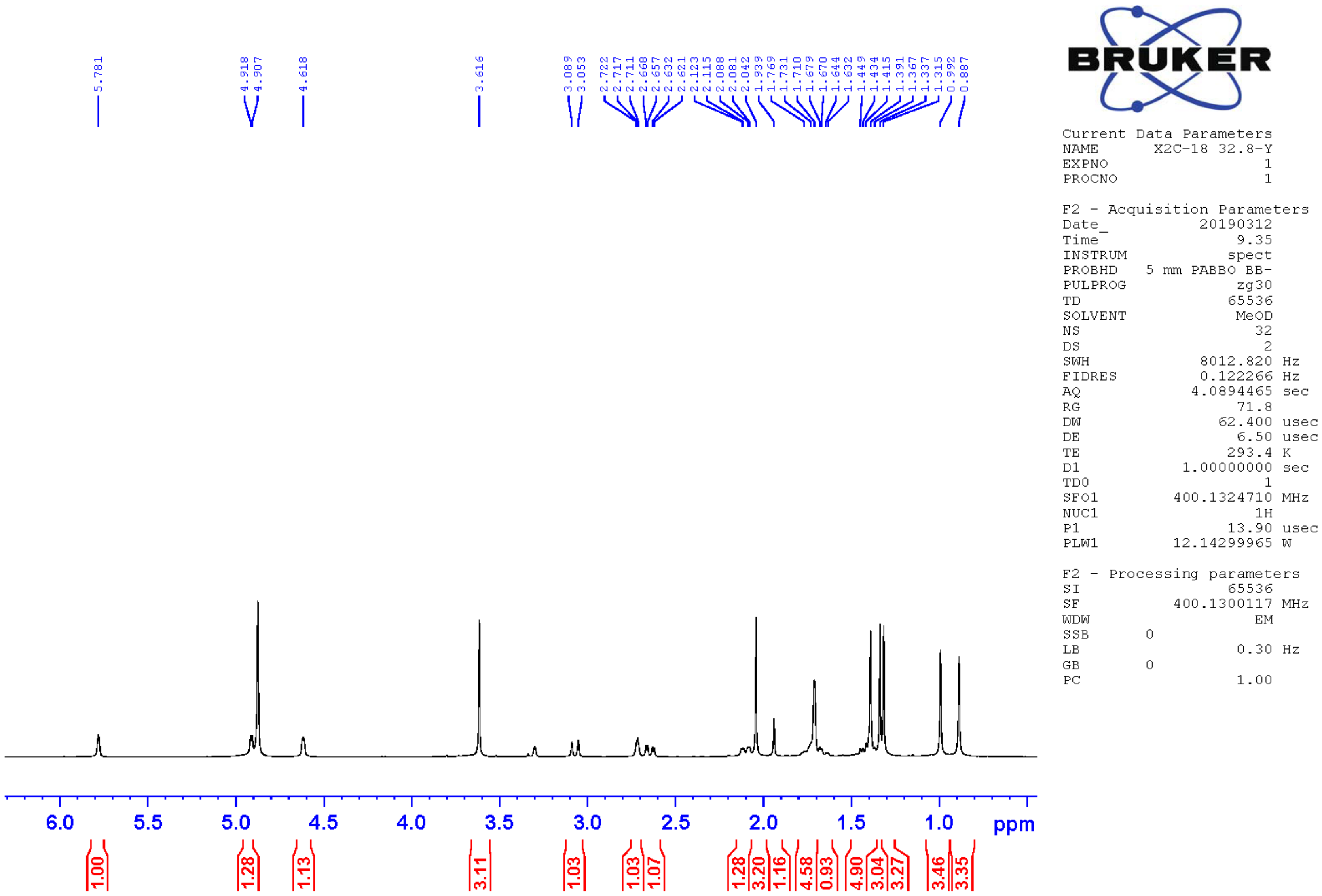

Supplementary Figure 13. ^{1}H NMR spectrum of **2** in CD_3OD.

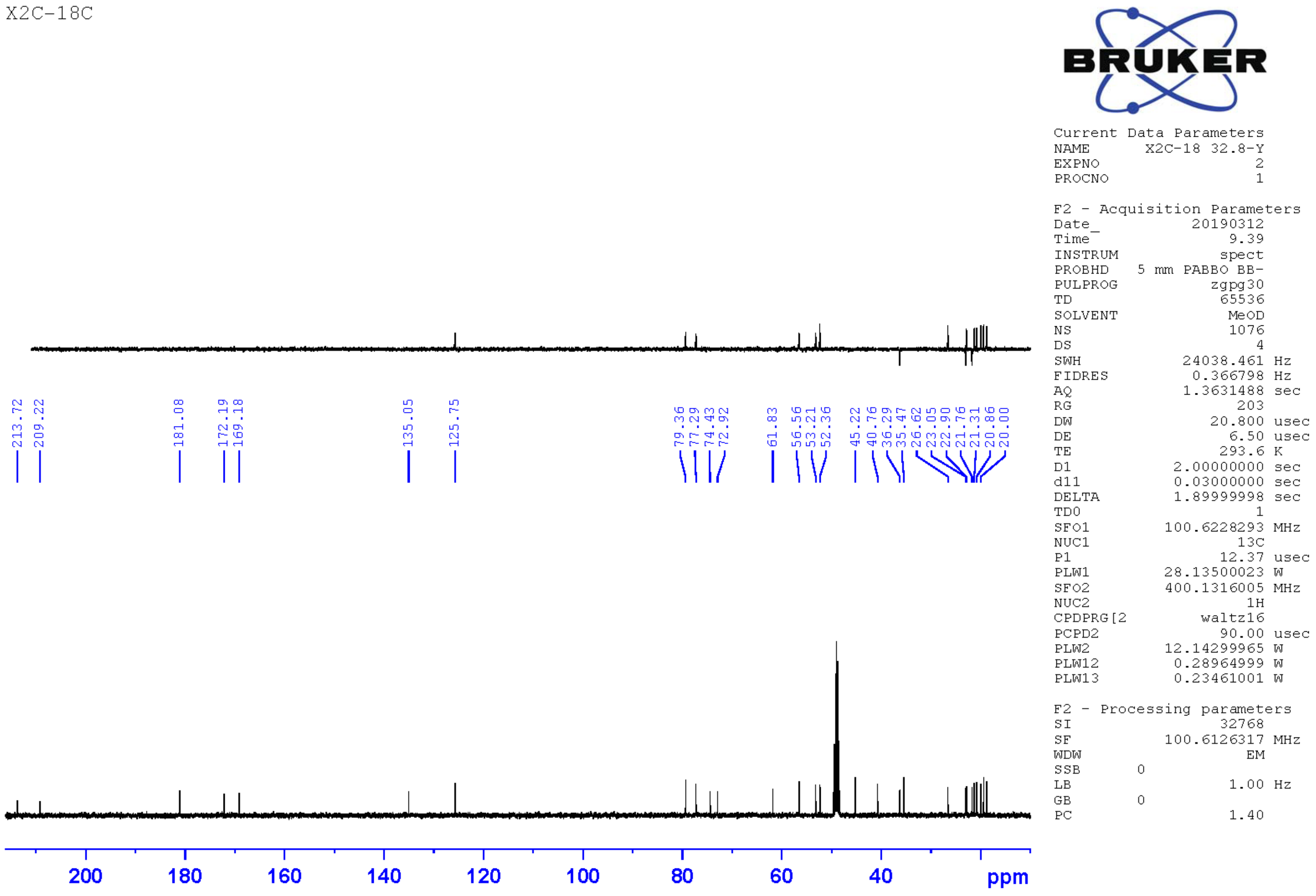

Supplementary Figure 14. ^{13}C NMR spectrum of **2** in CD_3OD.

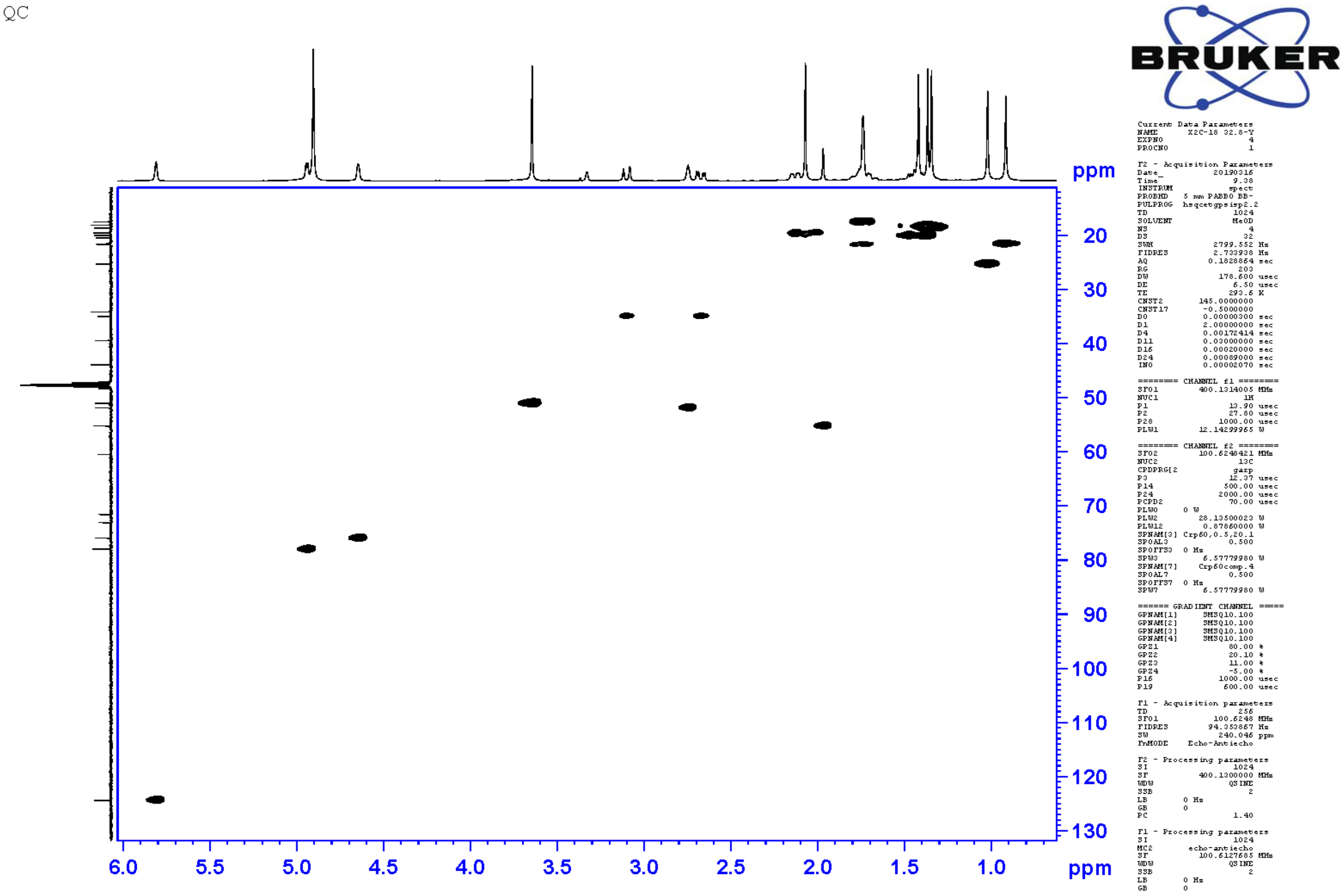

Supplementary Figure 15. HSQC NMR spectrum of **2** in CD_3OD.

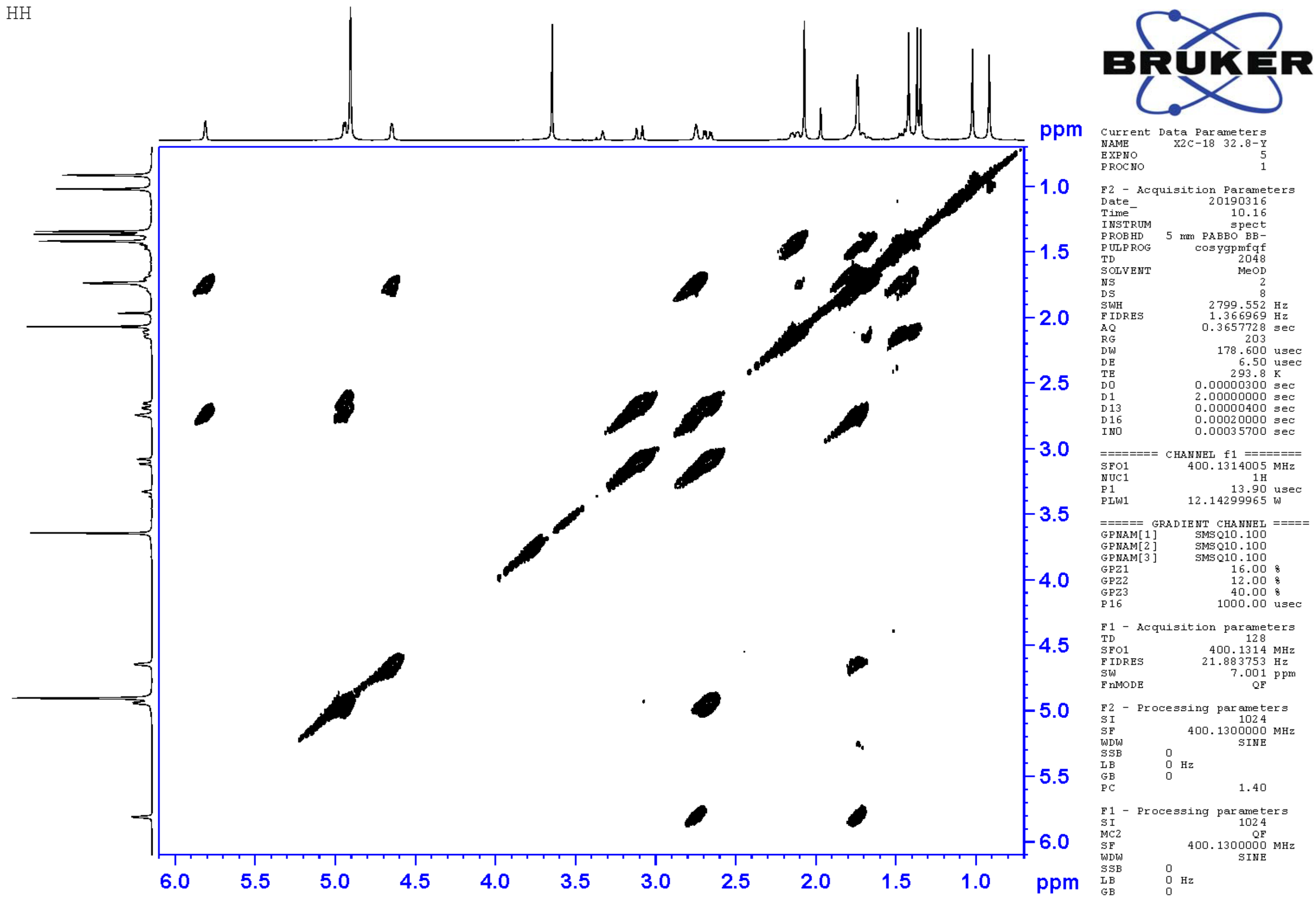

Supplementary Figure 16. COSY NMR spectrum of **2** in CD_3OD.

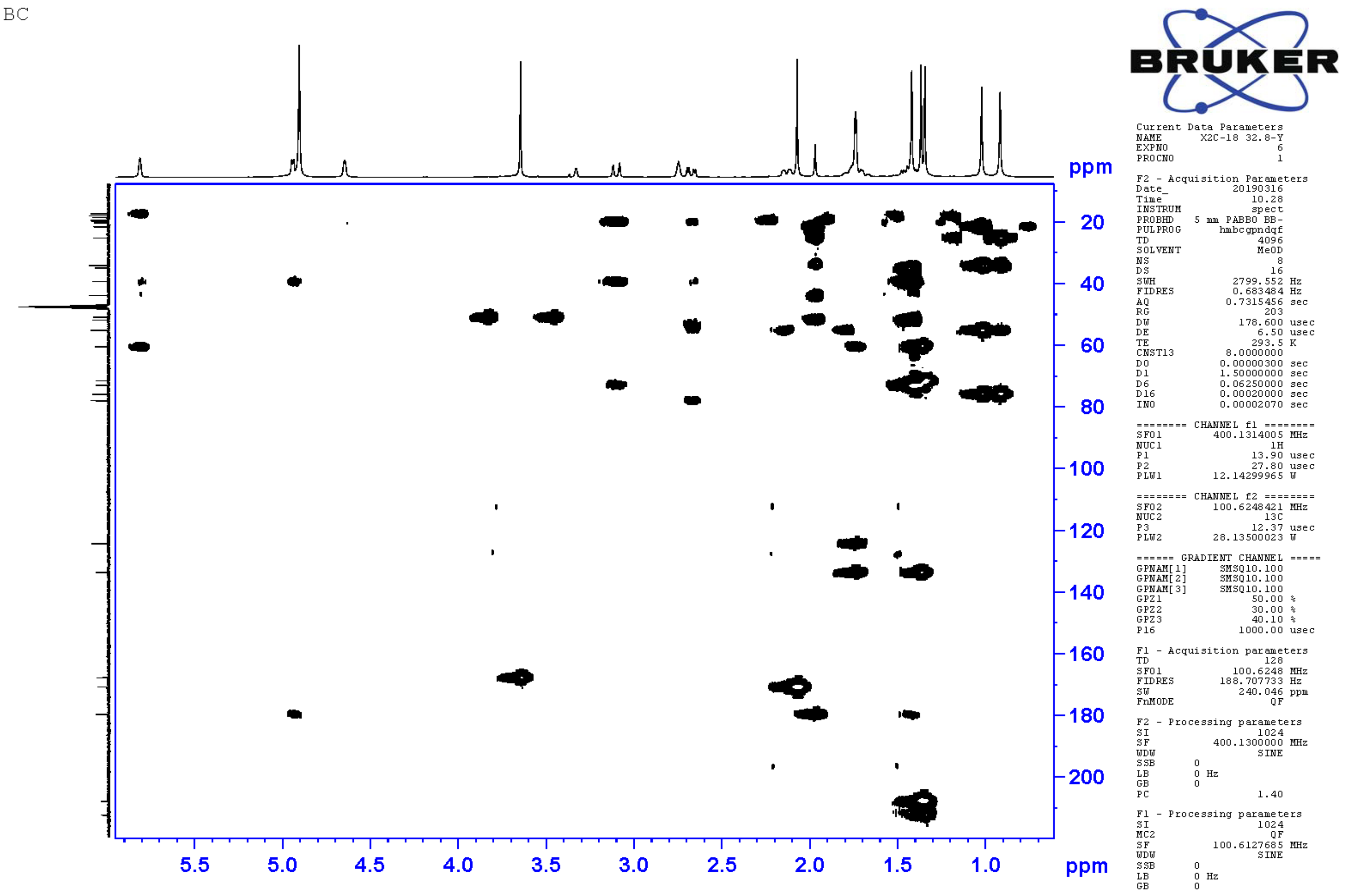

Supplementary Figure 17. HMBC NMR spectrum of **2** in CD_3OD
